# Local Vibrational Mode Analysis of Phonon Dispersion Relations in Crystals

**DOI:** 10.1021/acs.jctc.6c00097

**Published:** 2026-02-26

**Authors:** Mateusz Mojsak, Filippo Bodo, Alessandro Erba, Adam A. L. Michalchuk, Elfi Kraka

**Affiliations:** † School of Chemistry, 1724 University of Birmingham, Birmingham B15 2TT, United Kingdom; ‡ Dipartimento di Chimica, 9314 Universitá di Torino, via Giuria 5, 10125 Torino, Italy; § Department of Chemistry, 2765 Southern Methodist University, 3215 Daniel Ave, Dallas, Texas 75275-0314, United States; ∥ Federal Institute for Materials Research and Testing (BAM), Richard-Wilstaetter-Str 11, 12489, Berlin, Germany

## Abstract

We present a general framework for performing local vibrational mode analysis of vibrations in crystalline materials at arbitrary wavevectors throughout the Brillouin zone. The approach enables phonon dispersion relations to be interpreted in terms of chemically meaningful interatomic interactions and structural motifs, providing direct insight into the microscopic origins of the phonon behavior in periodic systems. We demonstrate the methodology for representative one-, two-, and three-dimensional materials including polymeric chains, graphene, and prototypical rock-salt and perovskite crystals. Across these systems, the analysis reveals how specific bonding patterns and structural features govern phonon dispersion relations. This framework provides a quantitative tool for the chemically intuitive analysis of phonon spectra and offers a pathway toward the rational design of phonon-dependent properties in crystalline materials.

## Graphical abstract

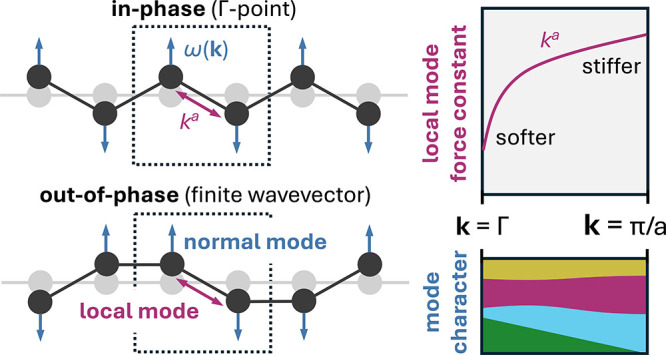

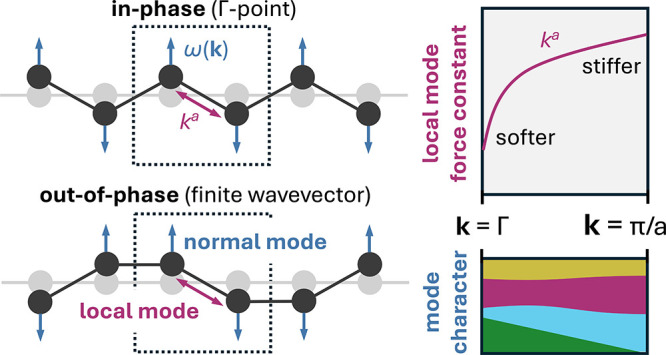

## Introduction

Vibrational spectroscopy provides detailed insight into the structure and dynamics of chemical systems and has become a core analytical tool in modern chemical science, frequently applied in both experiment and theory.^1–12^ Vibrational frequencies encode information on bond strengths and intermolecular interactions,^13–19^ while variations in vibrational spectra during chemical reactions or phase transformations offer insight into mechanisms of reactivity and structural response.^20–23^ In the solid state, structural (polymorphic) transformations are likewise reflected in the response of vibrational dynamics to perturbations such as temperature or pressure changes.^24–29^ As a result, vibrational spectroscopy is routinely used to characterize systems ranging from isolated molecules to extended crystalline solids, often supported and guided by theory.

Vibrational motion in chemical systems is commonly described in terms of normal modes, obtained by diagonalizing the mass-weighted force-constant matrix in Cartesian coordinates, which, for isolated molecular systems, is conventionally formulated within the Wilson GF formalism.^30^ In the harmonic approximation, normal modes form an orthogonal set whose equations of motion are dynamically decoupled in the normal-coordinate representation such that each mode behaves as an independent harmonic oscillator. This dynamical decoupling provides a mathematically simple and physically transparent framework that has enabled the broad application of normal-mode analysis across theoretical and computational chemistry, including the simulation of vibrational spectra, the computation of thermodynamic properties,^6,9,31–36^ the modeling of reaction pathways,^20,37,38^ and the description of phonon-mediated phenomena in solids.^24,39–41^

However, while the dynamical decoupling of normal vibrational modes underpins their mathematical and computational utility, their chemically intuitive interpretation at the atomic level remains a significant challenge.^12,42^ This difficulty arises because the displacement pattern associated with an individual normal vibrational mode typically corresponds to a collective, spatially delocalized motion involving many, or all, atoms in the system.^9,32,43^ Consequently, normal modes do not generally map in a straightforward manner onto chemically meaningful structural elements such as individual bonds, functional groups, or specific intermolecular interactions. For instance, it is often challenging to associate a given normal mode with a particular bond-stretching or angle-bending motion, which raises questions about the validity of relying on normal mode vibrational frequencies and force constants as measures of individual bond strengths.^44^ Moreover, a delocalized normal mode description can obscure intuitive structure–property relationships, e.g., how a particular bond, coordination motif, or local environment contributes to a vibrational feature or material response, thereby limiting the interpretability of vibrational spectra from a chemical perspective.

A natural route toward a more chemically intuitive description of vibrational motion is to express atomic displacements in terms of distortions of internal coordinates, such as bond stretches, angle bends, and torsions.^45–52^ The connection between internal coordinate distortions and Cartesian displacements is provided by the Wilson **B** matrix, which relates changes in internal coordinates to Cartesian displacements.^30,45,53–57^ Leveraging this internal coordinate representation of vibrational motion, we have developed local vibrational mode analysis (LMA). LMA extracts local vibrational modes and related properties from their delocalized normal vibrational mode counterparts.^13,15,58,59^

LMA has been successfully applied to characterize the strength of covalent bonds and noncovalent interactions across the periodic table and has been used to analyze molecular systems in the gas phase, solution, and protein environments. A comprehensive overview of local vibrational mode theory and its applications is available in recent review articles.^13,15^ More recently, LMA has been applied to characterize noncovalent interactions, including hydrogen bond strengths in protein–ligand complexes,^18^ weak and strong π interactions in sandwich complexes,^17,60^ and both metal–ligand and hydrogen bonding interactions in hemoglobins,^61,62^ cytochrome b5,^63^ and bacterioferritins.^64^ LMA has also found use in molecular and material design, for example to guide drug design,^65,66^ to develop a revised p*K*_a_ rule for the description of cocrystal formation,^67,68^ and to elucidate the vibronic coupling behavior in lanthanide complexes.^69–72^

Besides providing a quantitative measure of the intrinsic strength of a chemical bond or weak chemical interaction, another key feature of LMA is the characterization of normal modes (CNM) procedure.^73^ The CNM procedure decomposes each normal vibrational mode into local mode contributions, relying on an adiabatic connection scheme (ACS), which establishes a continuous mapping between a chemically motivated local mode description and the corresponding set of normal vibrational modes.^74^ By resolving normal modes into contributions from local vibrational coordinates through the CNM, the LMA framework enables quantitative structure–property relationships to be established at the level of individual bonds and interactions while remaining fully consistent with conventional vibrational theory. Both the CNM and ACS procedures, integrated in the LModeA software,^75–77^ have proven to provide a unique instrument for a comprehensive analysis and interpretation of vibrational spectra.^70,71,78,79^

LMA has been extended from molecular systems to periodic solids, enabling the analysis of periodic structures at the Brillouin zone center (**k** = **0**).^80^ LMA of these modes provides insight into overall bonding characteristics and enables comparison with experimental IR and Raman spectra.^81–83^ This extension has facilitated quantitative analysis of subtle changes in local bonding that arise from crystal packing, polymorphism, and external pressure. Applications include the *in situ* measurement of intrinsic bond strength in crystalline structures,^84^ the characterization of hydrogen bond variations across ice polymorphs,^22^ the tracking of local mode force constants from gas-phase to crystalline uranium-based systems,^80^ the prediction of impact sensitivities in energetic materials using the vibrational up-pumping model,^85^ and the elucidation of negative linear compressibility and phonon softening in Mg­(IO_3_)_2_ under pressure.^86^ Collectively, these studies show that crystal packing significantly influences local bonding, as reflected in local mode force constants, and leads to spectral shifts consistent with experimental observations. Furthermore, these studies establish LMA as a robust framework for linking local bonding descriptors to the vibrational properties in crystalline materials.

Though the study of Brillouin zone center vibrations in solids using LMA has proven extremely promising, many of the physical behaviors of solids depend on their dynamical behaviors away from **k** = **0**. In fact, vibrations at nonzero wavevectors play central roles in determining material properties like thermal conductivity,^87,88^ heat capacity,^89^ and phase stability,^90–92^ thereby defining material performance in applications such as thermoelectrics,^93^ and mechanical metamaterials.^94^ Furthermore, growing evidence suggests that phonon excitations and phonon scattering dynamics beyond the Brillouin zone center can influence reactive chemical processes in the solid state. For example, studies on mechanochemically reactive crystals indicate that nonequilibrium vibrational energy redistribution following the excitation of lattice vibrations may drive chemical reactivity.^95–98^ A chemically intuitive, local-coordinate-based interpretation of phonons across the Brillouin zone would therefore establish LMA as a powerful tool for crystal engineering and the rational design of functional materials based on chemically intuitive local structural descriptors; yet, it remains an outstanding challenge. Moreover, LMA in solids has so far focused primarily on obtaining bonding descriptors from local mode force constants, while the CNM in crystals has not yet been explored.

In this work, we derive a general formulation of LMA, including the CNM, to enable the analysis of arbitrary-wavevector vibrations in periodic solids while remaining consistent with the established molecular local mode framework. By constructing linearly independent sets of internal coordinates and extending the CNM procedure to finite wavevectors, we enable wavevector-resolved characterization of phonon modes in terms of chemically meaningful local distortions. We apply our methodology to representative one-, two-, and three-dimensional systems, demonstrating that our approach reveals how phonon dispersion, mode mixing, and branch connectivity arise from phase-adjusted local vibrations. This wave vector-resolved local mode perspective provides an intuitive, atomistic framework for interpreting phonon spectra and vibrational dynamics, bridging the gap between spectroscopic observables and chemical structure in extended systems (Figure 1).

**Figure 1:**
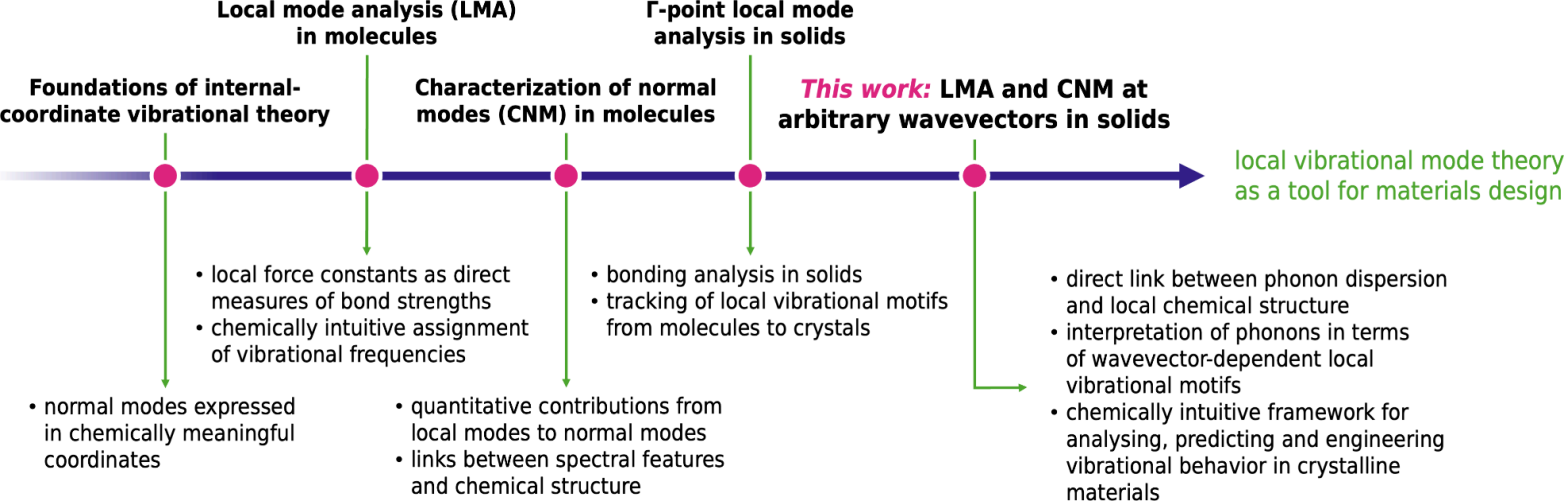
Evolution of local vibrational mode analysis and the characterization of normal modes. The scheme highlights major theoretical advances and their contributions to expanding the scope of chemically intuitive interpretations of vibrational dynamics. The present work adds a new step in this evolution, extending these concepts in the solid state beyond Γ-point vibrations. Our work enables the analysis of vibrations (phonons) across the Brillouin zone, enabling local-coordinate-based insight into phonon dispersion relations.

## Theoretical Background

The theoretical framework of LMA, originally introduced by Konkoli, Cremer, and co-workers,^58,59,99,100^ has been continuously developed over the past two decades. Rigorous derivations and a comprehensive presentation of the underlying local vibrational mode theory can be found in two review articles.^13,15^ Here, we do not reproduce these derivations in full. Instead, we briefly summarize the key elements of LMA required to establish notation, clarify the physical interpretation, and provide a consistent starting point for the developments presented in this work. Building on this foundation, we then introduce the physical rationale, mathematical formulation, and practical aspects of our generalization of LMA to arbitrary wavevectors in periodic systems.

### Local Vibrational Mode Theory for Isolated Molecules

#### Normal Modes in Isolated Molecules

In harmonic vibrational analysis, a molecule’s vibrational behavior is modeled from the curvature of its potential energy surface about its equilibrium geometry. This curvature is encoded in the force constant matrix, **f** ^*x*^, which describes how the electronic potential energy of the system changes in response to small displacements of the nuclei. We use the superscript *x* to indicate that the matrix is expressed in Cartesian coordinates. The elements of **f** ^*x*^ are the second derivatives of the total electronic energy with respect to Cartesian coordinates *x*_κα_,

1 $$f_{\kappa \alpha ,\kappa \prime \alpha \prime }^{x}=\frac{\partial^{2}E}{\partial x_{\kappa \alpha }\partial x_{\kappa \prime \alpha \prime }}$$

and they quantify the energy associated with the displacement of atoms κ, κ′ (with κ = 1, ..., *N*_atoms_) along Cartesian directions α, α′ ∈ {*x*, *y*, *z*}. The set of *N*_*Q*_ = 3*N*_atoms_ *normal vibrational modes* and their corresponding harmonic frequencies is obtained by solving the generalized eigenvalue problem for vibrational motion,^101^

2 $$f^{x}L=\mathrm{ML}\Lambda$$

where the columns **l**_μ_ of the *N*_*Q*_ × *N*_*Q*_ matrix **L** store the normal mode eigenvectors in Cartesian coordinates. The nonzero diagonal elements of **Λ** are the eigenvalues λ_μ_ = ω_μ_^2^ that are related to the squared harmonic normal-mode frequencies ω_μ_. The 3*N*_atoms_ × 3*N*_atoms_ diagonal matrix **M**, defined by *M*_κα,κ′α′_ = δ_κα,κ′α′_ *m*_κ_, introduces the correct inertial weighting that arises from the atomic masses *m*_κ_. By convention, in the isolated molecular LMA formalism, the eigenvectors that correspond to zero eigenvalues, i.e., those that describe motions under which the potential energy is invariant, are removed.^13^ This convention restricts the analysis to the subspace of internal vibrations, with *N*_vib_ = *N*_*Q*_ – 6 eigenvectors and eigenvalues in nonlinear molecules, or *N*_vib_ = *N*_*Q*_ – 5 in linear molecules.

The force constant matrix **f** ^*x*^ can be transformed into the normal coordinate basis as,

3 $$f^{Q}=K=L^{T}f^{x}L$$

where **f** ^*Q*^ = **K** is an *N*_vib_ × *N*_vib_ diagonal matrix of force constants expressed in terms of normal coordinates *Q* = (*Q*_1_, *Q*_2_, ..., *Q*_*N*_vib__). The basis of normal coordinates represents the vibrational dynamics in terms of independent modes of collective motion of all atoms, such that the vibrational modes are decoupled in the harmonic approximation, where each *Q*_μ_ represents the amplitude of atomic motion along the corresponding normal mode **l**_μ_.

#### Local Modes and Their Properties for Isolated Molecules

Due to their delocalized nature,^9,32,43^ normal modes provide limited insight into how specific chemical interactions, such as individual bonds, angles, or torsions, participate in the vibration described by a given eigenvector **l**_μ_. To establish a direct link between vibrational motion and chemically intuitive structural units, local vibrational mode theory re-expresses vibrational dynamics in terms of displacements along specific internal coordinates associated with the structural units under consideration. In this respect, Konkoli and Cremer introduced *adiabatic internal coordinate modes* by initiating motion along a single internal coordinate and allowing all remaining coordinates to relax self-consistently.^58,59^ Formally, the resulting displacement pattern follows from mass-decoupled Euler–Lagrange equations in which only the masses of the associated atomic fragment are nonzero. The procedure yields *local vibrational modes* that are decoupled from the rest of the system and retain a strict one-to-one correspondence with individual structural features. In contrast to delocalized normal modes, these local modes encode the topology of the potential energy surface in the immediate vicinity of the selected internal coordinate and hence enable the assignment of force constants, frequencies, and energy distributions to specific bonds or torsions. By making such local assignments, we obtain a means to quantify the intrinsic strength and vibrational dynamics of individual chemical interactions.

As in normal mode theory, the relationship between internal coordinates *q* and Cartesian displacements in local vibrational mode theory is described by the Wilson **B** matrix, a rectangular matrix of size *N*_int_ × 3*N*_atoms_, where *N*_int_ is the number of internal coordinates. Each element *B*_*n*,κα_ is defined as the partial derivative of the *n*-th internal coordinate *q*_*n*_ with respect to the Cartesian coordinate *x*_κα_,^101^

4 $$B_{n,\kappa \alpha }=\frac{\partial q_{n}}{\partial x_{\kappa \alpha }}$$

Hence, any structural feature for which (1) a value can be assigned based on the geometry of the studied system and (2) a gradient with respect to the displacements of the involved atoms can be obtained, either analytically or numerically, may be used as an internal coordinate for LMA.^13,15,17^

The **B** matrix maps the normal mode eigenvectors, **L**, expressed in Cartesian coordinates, onto the internal coordinate representation,

5 $$D=\mathrm{BL},\mathrm{such},\mathrm{that},D_{n,\mu }=\underset{\kappa \alpha }{\sum }\frac{\partial q_{n}}{\partial x_{\kappa \alpha }}l_{\mu ,\kappa \alpha }$$

where the rows **d**_*n*_ store the contributions of internal coordinate *q*_*n*_ to the normal modes. The kinetic energy metric in the internal coordinate space is given by the Wilson G matrix,

6 $$G={\mathrm{BM}}^{-1}B^{T},\mathrm{such},\mathrm{that},G_{n,n\prime }=\underset{\kappa \alpha }{\sum }\frac{1}{m_{\kappa }}\frac{\partial q_{n}}{\partial x_{\kappa \alpha }}\frac{\partial q_{n\prime }}{\partial x_{\kappa \alpha }}$$

which defines the pairwise mass-weighted coupling between displacements along internal coordinates.^102^ The local mode adiabatic vectors in Cartesian coordinates, **a**_*n*_^*x*^, are given by

7 $$a_{n}^{x}=L\cdot \frac{K^{-1}d_{n}^{T}}{d_{n}K^{-1}d_{n}^{T}}$$

The local mode force constants are given by

8 $$k_{n}^{a}={\left(d_{n}K^{-1}d_{n}^{T}\right)}^{-1}$$

and have the dimension [*k*_*n*_] = *M*·*T* ^–2^·[**B**_*n*_]^2^. Correspondingly, for internal coordinates with nondimensionless derivatives (e.g., angles and dihedrals), they need to be normalized to yield an effective force constant, *k*_*n*_^eff^, with the correct dimensions.^13^ The local mode eigenvalues (squared angular frequencies) are given by

9 $${\left(\omega_{n}^{a}\right)}^{2}=G_{n,n}\times k_{n}^{a}$$

where the diagonal elements of the **G** matrix return the mass weighting.^103^

#### Characterization of Normal Modes

Within the framework of CNM,^73,76,77^ the relationship between normal modes and local vibrational modes is quantified by evaluating a force-constant-weighted overlap between normal mode eigenvectors and the local mode adiabatic vectors. For any pair of normal mode eigenvector **l**_μ_ and local mode adiabatic vector **a**_*n*_^*x*^, a quantitative comparison can be defined by means of an amplitude, *A*_*n*,μ_, which facilitates the extraction of chemical information.^104^ A common definition of the amplitude, interpreted as the degree to which “normal mode **l**_*μ*_ originates electronically from the internal vibration **a**_*n*_^*x*^”,^105^ is given by

10 $$A_{n,\mu }=\frac{\left\langle l_{\mu }\right|f^{x}\left|a_{n}^{x}\right\rangle }{\left\langle l_{\mu }\right|f^{x}\left|l_{\mu }\right\rangle \left\langle a_{n}^{x}\right|f^{x}\left|a_{n}^{x}\right\rangle }=\frac{|D_{n,\mu }{|}^{2}\times k_{n}^{a}}{K_{\mu ,\mu }}$$

The local-mode character of the normal mode μ in terms of **a**_*n*_^*x*^ can be expressed as a fraction of the corresponding amplitude over the sum of *A*_*n*,μ_ calculated with respect to this normal mode’s eigenvector,

11 $$C_{n,\mu }=\frac{A_{n,\mu }}{\overset{3N}{\underset{n^{\prime }}{\sum }}A_{n^{\prime },\mu }}$$

We note, however, that care must be taken when interpreting the local-mode characters computed as *C*_*n*,μ_. The adiabatic vectors that are computed for local modes with chemically meaningful internal coordinates generally do not form an orthogonal basis. Correspondingly, some overlap between the contributions of these local modes to the normal modes is unavoidable. Hence, the normal mode character descriptors *C*_*n*,μ_ should be interpreted as a *relative similarity metric* between a given normal and local mode, rather than as measure of the *composition* of the normal mode in terms of the set of local modes.

### Local Vibrational Mode Theory in Periodic Systems

The structure of a periodic system is defined by a single repeat unit—the unit cell, translated by lattice vectors **R**—while its vibrational dynamics involve the relative motion of atoms in neighboring cells. Accordingly, vibrational modes in a periodic system are described using both the displacement pattern of the normal mode eigenvector within the unit cell, **L**(**k**), and a Bloch phase factor that accounts for its spatial propagation through the lattice. Crucially, both terms depend on the wavevector **k**, such that the displacement of atom κ at position **R** that arises from normal mode μ at wavevector **k** is given by

12 $$u_{\kappa }\left(R\right)=l_{\kappa \mu }\left(k\right)\times e^{ik\cdot R}$$

In the harmonic approximation, the wavevector-dependent normal mode eigenvectors are conventionally obtained by diagonalizing the mass-weighted dynamical matrix **W**(**k**), whose elements are obtained as

13 $$W_{\kappa \alpha ,\kappa \prime \alpha \prime }\left(k\right)=\frac{1}{\sqrt{m_{\kappa }m_{\kappa \prime }}}\underset{R_{\lambda }}{\sum }\frac{\partial^{2}E}{\partial x_{\kappa \alpha }^{0}\partial x_{\kappa \prime \alpha \prime }^{R_{\lambda }}}\times e^{ik\cdot R_{\lambda }}$$

where **R**_λ_ is a lattice vector from the reference unit cell at **R** = **0** to the λ-th periodic image. Importantly, the eigenvectors are uniquely defined for a given wavevector and must be explicitly calculated at each **k**.

Hence, to perform wavevector-resolved LMA in a periodic system, we must first compute **W**(**k**) at the appropriate wavevector, eq 13, with which we can obtain the equivalent of the **f** ^*x*^ matrix, eq 1, by removing its mass weighting,

14 $$f^{x}\left(k\right)=M^{1/2}W\left(k\right)M^{1/2}$$

where **M** is a 3*N*_atoms_ × 3*N*_atoms_ matrix containing the masses of all atoms in the unit cell of the periodic system. The eigenvectors **L**(**k**) and eigenvalues **K**(**k**) used as inputs for our LMA framework are obtained by solving the generalized eigenvalue problem, eq 2, involving **f** ^*x*^(**k**) .

The matrix **f** ^*x*^(**k**) is Hermitian and complex-valued in general, though the imaginary component vanishes at **k** = Γ and at the edge of the first Brillouin zone. Consequently, eqs 2 and 3 and 5–11 can be used for LMA and CNM in periodic systems at any wavevector using the same formalisms derived for isolated molecules (see Local Vibrational Mode Theory for Isolated Molecules), so long as (1) the transpose operations are replaced with the Hermitian transpose to appropriately handle complex-valued matrices, and (2) the following challenges specific to periodic systems are addressed:

- Internal coordinate definitions must account for periodic boundary conditions and structural symmetries.
- Local mode atomic displacements must obey the correct phase relationships imposed by **k** ≠ **0** wavevectors.

In our earlier extension of LMA to periodic systems at **k** = **0**, the first challenge was partially addressed for bond internal coordinates.^106^ More generally, the construction of internal coordinate representations for periodic systems has been considered previously in the context of geometry optimization. For example, internal coordinate representations have been central to the development of optimization algorithms that are based on redundant internal coordinates.^107–109^ In the present paper, we extend the LMA framework to include the definition of *arbitrary* internal coordinates in periodic systems, within a vibrational analysis context. The second challenge did not arise previously.

In the following sections, we present generalized solutions to both challenges. These developments yield a local mode formalism for periodic systems that is fully consistent with the approach for isolated molecular systems while enabling a complete local mode analysis of phonon dispersion behavior.

#### Internal Coordinates and the **B**-Matrix for Periodic Systems

Defining unique internal coordinates in isolated molecules is relatively straightforward as all internal coordinates are fully specified by the structural model. In contrast, the unit cell description of periodic systems implies that some internal coordinates are not confined to a single unit cell but instead involve atoms that span periodic boundaries. Moreover, in small network systems, some internal coordinates may even involve periodic images of the same atom. These features make the definition of a unique set of internal coordinates a particular challenge in periodic systems. Nevertheless, these challenges can be systematically addressed for an arbitrary choice of internal coordinates as follows:

1. Construct a cluster around a reference unit cell by applying lattice translation vectors to the positions of atoms in the reference cell. The cluster should be sufficiently large such that each internal coordinate of interest is represented at least once.
2. Based on the atoms within the cluster, define internal coordinates relevant to the chemical problem under study. Alternatively, an automatic graph-theoretic search (details in ref 110 and ESI, S1) can be used to identify all internal coordinates of a given type (including, but not limited to bonds, angles, and dihedrals) in the cluster.
3. If multiple periodic images of the same internal coordinate are generated in step 1, select a unique representation by choosing the image for which a designated reference atom lies within the reference unit cell, Figure 2. This ensures that copies of the same coordinate that are related by lattice translations are not included multiple times. While the choice of the reference atom does not affect the computed local mode properties, it must be chosen consistently across all internal coordinates of the same type, see ESI, S1.
4. Reduce the internal coordinate set to a minimal linearly independent set that spans the vibrational space of the system. This can be achieved by performing a QR decomposition to retain only linearly independent rows in the **B** matrix, while allowing any selected internal coordinate to be preserved. Removing linear dependencies from the internal coordinate set guarantees that the similarity metrics computed in the CNM procedure capture distinct information about the normal mode characters, albeit subject to some overlap due to the nonorthogonality of local modes.

**Figure 2:**
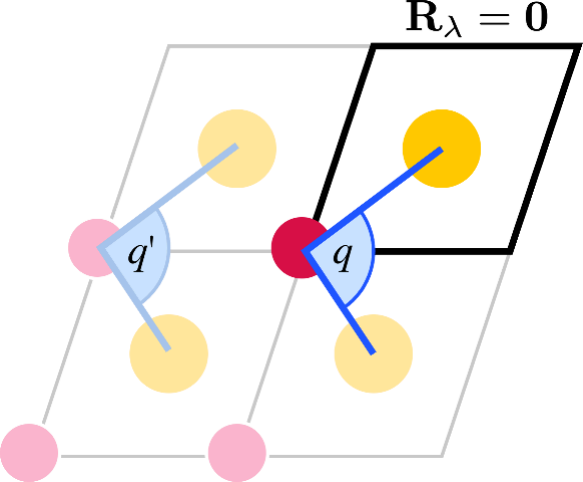
An example cluster constructed as a 2 × 2 supercell around a reference unit cell, top right, which contains two atoms: red and yellow. The internal angle coordinate *q* crosses the unit cell boundary and involves two periodic images of the same atom (yellow). As defined, the cluster contains two equivalent periodic images of *q*. A unique representation can be found by only considering the case where the internal coordinate’s central atom (red) belongs to the reference unit cell.

Having chosen an appropriate set of internal coordinates for the periodic system, one must subsequently construct the **B** matrix. Importantly, the **B** matrix must be defined with respect to all of the atoms participating in the chosen set of internal coordinates. Consequently, to compute the coupling between internal coordinates and normal modes (eq 5), the normal coordinate representation must also include all atoms associated with the internal coordinates identified in the above procedure. Standard definitions of normal modes in periodic systems (eq 13) describe displacement patterns for only atoms within a single unit cell. The displacement of atoms in all other cells is instead defined implicitly through translational symmetry and the associated Bloch phase factors. This presents a challenge when considering internal coordinates that cross unit cell boundaries and hence include atoms that are not explicitly contained in the normal coordinate definition. This issue is particularly significant for LMA performed at **k** ≠ **0**, where the relative phase of atomic displacements in neighboring unit cells must be explicitly accounted for when evaluating the derivatives of internal coordinates that are required to construct the **B** matrix. Moreover, when internal coordinate definitions involve periodic images of the same atom, the corresponding derivatives with respect to atomic positions are inherently coupled, as the displacements of periodic images are related through the Bloch phase factor.

We present here two approaches to overcome these issue, the Expanded **B**-Matrix Method, and the **k**-Dependent **B**-Matrix Method.

##### Expanded **B**-Matrix Method

The internal coordinates and their phase relationships across periodic boundaries can be treated explicitly. To do so, the **B** matrix is constructed to capture all *N*_clst_ atoms that were present in the cluster used to identify the internal coordinates, above. This expanded matrix B* has dimensions *N*_int_ × 3*N*_clst_, as it contains additional columns for the internal coordinate derivatives with respect to atoms that are formally outside the reference unit cell. Each element of an expanded row **b**_n_^*^ is evaluated independently, and is given by

15 $$b_{n,\lambda \kappa \alpha }^{*}=\frac{\partial q_{n}}{\partial x_{\lambda \kappa \alpha }}$$

where the index λ differentiates atoms which formally belong to different unit cells, and the asterisk indicates a vector that has been expanded to describe the cluster.

To construct the **D** matrix, the columns of the eigenvector matrix **L**(**k**) must also be expanded such that they describe the displacement pattern of all atoms in the cluster, taking the shape 3*N*_clst_ × *N*_*Q*_. To ensure displacement patterns respect the appropriate phase relations, we generate the displacement patten for atoms outside the reference cell according to eq 12, with the elements of the expanded eigenvector **l**_μ_^*^(**k**) for the normal mode μ at wavevector **k** given by

$$l_{\lambda \kappa \alpha ,\mu }^{*}\left(k\right)=l_{\kappa \alpha ,\mu }\left(k\right)e^{ik\cdot R_{\lambda }}$$ 16

It follows that the elements of the **D** matrix at wavevector **k** are given by

17 $$D_{n,\mu }\left(k\right)=b_{n\cdot }^{*}\cdot l_{\cdot \mu }^{*}\left(k\right)=\underset{\lambda \kappa \alpha }{\sum }\left(\frac{\partial q_{n}}{\partial x_{\lambda \kappa \alpha }}l_{\kappa \alpha ,\mu }\left(k\right)e^{ik\cdot R_{\lambda }}\right)$$

and the matrix retains the *N*_int_ × *N*_*Q*_ dimension under row × column multiplication. Similarly, the **G** matrix retains the correct *N*_int_ × *N*_int_ dimension, with the summation in eq 6 now also running over the unit cell index λ. Hence, after (1) expanding the **B** matrix and (2) expanding and phase-adjusting the eigenvector matrix **L**(**k**), the **k**-dependent local mode properties and normal mode characters can be computed in the same way as for isolated molecular systems (eqs 7–11, using the Hermitian transpose).

##### **k**-Dependent **B**-Matrix Method

The Expanded **B**-Matrix Method provides an intuitive, explicit handling of the phase relationships that are needed to derive local modes at **k** ≠ **0** wavevectors in periodic systems. However, the method relies on generating modified eigenvector matrices for the LMA for each wavevector being studied.

When handling internal coordinates that span unit cell boundaries, the Expanded **B**-Matrix Method introduced the phase relationship by explicitly adding additional atoms to the normal mode eigenvector descriptions, **L**(**k**) . However, rather than expanding the **B** and **L**(**k**) matrices to accommodate *N*_clst_ atoms, we can instead subsume the phase relation directly into our **B** matrix definition, see ESI, S2.1, such that

18 $$B_{n,\kappa \alpha }\left(k\right)=\underset{\lambda }{\sum }\frac{\partial q_{n}}{\partial x_{\lambda \kappa \alpha }}e^{ik\cdot R_{\lambda }}$$

We note that for local modes that are contained entirely within the unit cell (i.e., only contain atoms of **R**_λ_ = **0**) eq 18 reduces to the standard definition, eq 4.

This approach yields equivalent results to the expanded **B** matrix method, see ESI, S2.2, with the added benefit of requiring no modification to the size of the **B** and **L**(**k**) matrices. This approach provides access to a **k**-dependent **B**(**k**) matrix that respects the periodic boundary conditions at any wavevector for which normal mode eigenvectors have been calculated. Moreover, by encoding the phase relationships directly in the **B**(**k**) matrix, eq 18, our method becomes analogous to the original molecular formalism of LMA, wherein the **B** matrix, acting on Cartesian normal mode eigenvectors, gives the transformation of normal coordinates to internal coordinates.

The diagonal elements of the **G** matrix, eq 6, that are used to obtain local mode frequencies, eq 9, are identical when generated by both approaches.

#### Interpreting Local Modes in Periodic Systems

In the LMA framework,^102^ local modes are defined as vibrations that reflect the internal distortions of a molecule. Consequently, normal modes that leave the molecular structure undistorted—namely rotations and translations, are conventionally excluded from local mode analyses, since they do not contribute to the internal vibrational dynamics of the molecule and have no direct connection to experimental observables. Similarly, in the Γ-point formalism for LMA in periodic solids by Bodo et al.,^86^ the eigenvectors that correspond to the three translational (and one rotational in 1D systems) normal modes are excluded, restricting the analysis to vibrations that distort the crystal structure and a set of 3*N*_atoms_ – 3 or 3*N*_atoms_ – 4 strictly positive eigenvalues.^106,111^

At a nonzero wavevector, however, even when infinitesimally close to **k** = **0**, the translational and rotational modes in solids become flexing or twisting motions, which distort the crystal structure and impact its internal energy. This leads to additional modes with nonzero eigenvalues, which describe physically relevant vibrations of the system. Hence, with our goal of analyzing the local mode contributions to phonon branches as they vary with wavevector, we must be able to smoothly transition from Γ-point to finite-wavevector phonons, applying our formalism to a vibrational space described by a set of eigenvectors of consistent size. Hence, for a generalized, arbitrary-wavevector description of LMA, the full set of 3*N*_atoms_ eigenvalues and eigenvectors must be retained across the Brillouin zone. This introduces two challenges: (1) the translational and rotational motions have no representation in conventional internal coordinates and (2) the presence of zero eigenvalues at the Γ-point leads to singular expressions in eqs 7–11.

To address the first challenge, we propose that three rigid-body translation “internal coordinates” be included in the analysis, corresponding to the displacements of all atoms in the *x*, *y*, and *z* Cartesian directions. Furthermore, for 1D systems, an additional coordinate should be included, corresponding to the rotational “internal coordinate” around the axis aligned parallel to the periodic direction. The **B** matrix elements for these special coordinates for atom κ take the following forms:

19 $$\begin{array}{c} 3D,2D\mathrm{and}1D,\mathrm{systems}:\\ \mathrm{translation along}x:\frac{\partial q_{x}}{\partial r_{\kappa }}=\left(1,0,0\right),\\ \mathrm{translation along}y:\frac{\partial q_{y}}{\partial r_{\kappa }}=\left(0,1,0\right),\\ \mathrm{translation along}z:\frac{\partial q_{z}}{\partial r_{\kappa }}=\left(0,0,1\right),\\ 1D\mathrm{systems only}(\mathrm{if periodic axis}=z):\\ \mathrm{rotation around}z:\frac{\partial q_{z}}{\partial r_{\kappa }}=\left(-y_{\kappa },x_{\kappa },0\right) \end{array}$$

The inclusion of these special coordinates in the internal coordinate set ensures that, at the Γ-point, the CNM procedure identifies the acoustic modes as being characterized by those rigid body translations or rotations, and the changing character of the acoustic branches at finite wavevectors is smoothly captured by the growing contributions from the remaining local modes.

To address the issue of undefined local mode properties at the Γ-point when zero eigenvalues are explicitly included, one must regularize these eigenvalues by replacing exact zeroes with infinitesimal positive values. This procedure ensures matrix invertibility while preserving the full orthogonality and completeness of the normal mode basis for local mode analysis across all wavevectors. Notably, this procedure has no physical impact on the nonzero spectrum or resulting local mode properties (ESI, S3).

It is important to emphasize that once the local modes are defined according to our formalism, the local mode force constants at a particular wavevector reflect the system’s stiffness in response to distortions of the internal coordinates, obeying the phase relations dictated by that wavevector. As such, these local force constants are not a direct measure of intrinsic bond strengths in the conventional sense. Nevertheless, these local mode properties remain physically meaningful: they directly encode the contributions of local structural motifs to phonon dispersion, providing a chemically intuitive lens through which to interpret how collective vibrations emerge from the underlying interactions in the material.

#### Summary of Proposed Method

Applying the formalism described above allows for the analysis of local modes in periodic solids subject to vibrations under any wavevector. The key steps in our generalized approach for local mode analysis in solids and the link of our proposed extension to established local vibrational mode theory are summarized in Figure 3. In the following sections, we demonstrate the applications of our new formalism to several model systems.

**Figure 3:**
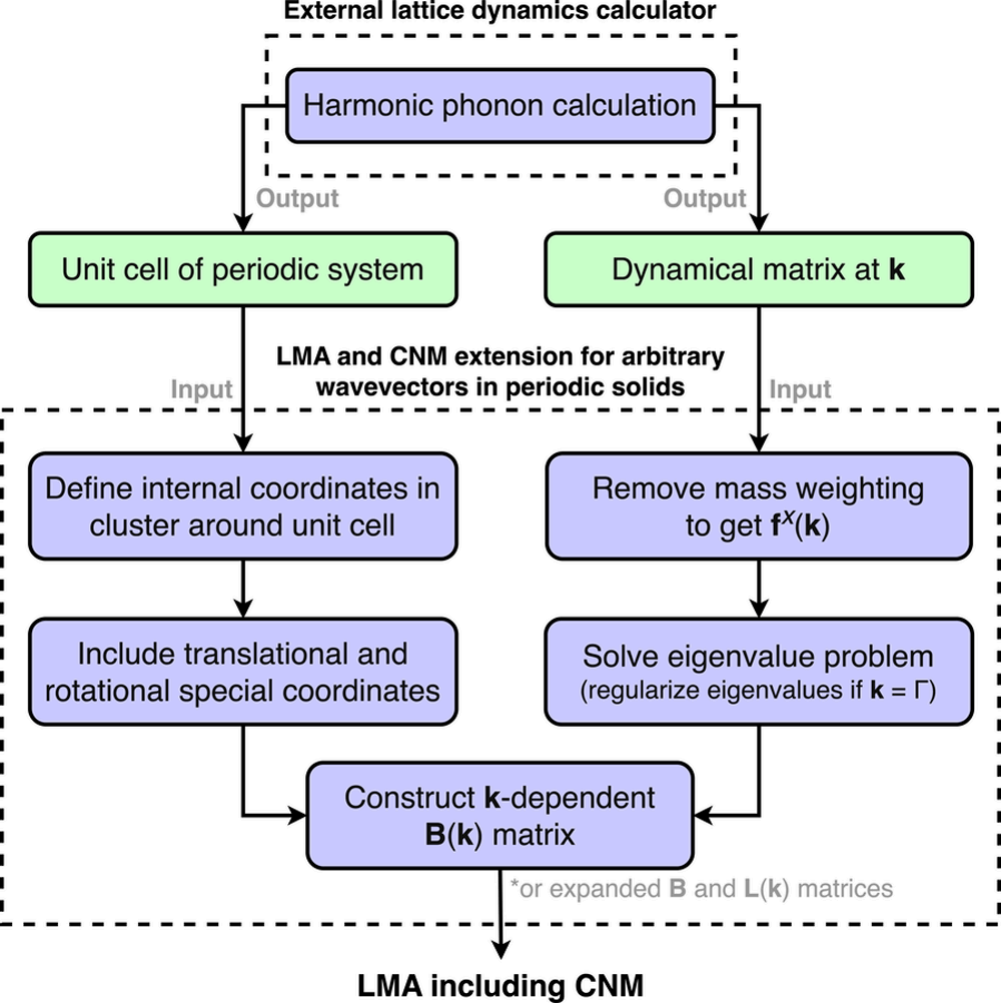
Summary of the arbitrary-wavevector extension to the local vibrational mode theory for periodic systems using the Expanded **B**-Matrix Method. The flowchart shows how the two inputs necessary to perform LMA and CNM in periodic systems, the optimized unit cell and the dynamical matrix computed for a given wavevector, are processed according to our method. Once the **k**-dependent **B**(**k**) matrix is computed, LMA and CNM can be performed according to the conventional formalism, eqs 5–11.

## Computational Methods

All calculations used as inputs for local mode analysis in this manuscript were performed within the framework of plane-wave density functional theory (DFT) as implemented in CASTEP v25.11.^112^ Crystal structures of our three-dimensional model systems magnesium oxide and potassium magnesium fluoride were obtained from the Inorganic Crystal Structure Database (ICSD) with collection codes 159375 ^113^ and 40477,^114^ respectively. The structure of graphene was obtained from the Materials Project for C (mp-990448) from database version v2025.09.25,^115^ and included an 8.52 Å vacuum layer in the *z*-direction to model the system as being effectively two-dimensional. The structures of *cis*- and *trans*-polyacetylene were custom-built, and included a 10 Å vacuum layer in the *x*- and *y*-directions to model the system as being effectively one-dimensional. Input and optimized atomic coordinates are given in ESI, S4.

For all systems, input structures were relaxed with the exchange-correlation energy described using the generalized gradient approximation (GGA) functional of Perdew–Burke–Ernzerhof (PBE).^116^ The Grimme D2 dispersion correction^117^ was used in models of all systems except graphene, where the dispersion correction was not included to avoid modeling interlayer interactions. The nuclear Coulomb potential was attenuated with norm-conserving pseudopotentials as obtained on-the-fly within CASTEP. The wave function was expanded in a plane-wave basis set to a kinetic energy cutoff of 1200 eV, and sampled on a Monkhorst–Pack k-point grid with spacing of 0.05 Å^–1^. Convergence criteria for the self-consistent field cycles were set to an electronic energy change <10^–10^ eV and an electronic eigenvalue change <10^–12^ eV. The Broyden density-mixing scheme was used to accelerate SCF convergence, with a mixing amplitude of 0.5 and a maximum mixing G-vector of 1.5 Å^–1^. Structure relaxation used the limited-memory Broyden-Fletcher-Goldfarb-Shanno (LBFGS) algorithm,^118^ with convergence reached when the total energy change was <2 × 10^–6^ eV atom^–1^, residual forces <10^–4^ eV Å^–1^, ionic displacements <10^–5^ Å and cell strain <0.1 GPa.

Phonon calculations were performed using the linear response method, as implemented in CASTEP v25.11,^112^ with LO-TO splitting enabled for the three-dimensional model systems, and disabled for the other (nonpolar) systems. To compute phonon dispersion relations, Brillouin zones of the model systems were sampled on Γ-centered Monkhorst–Pack grids with *ca*. 0.05 Å^–1^ k-point spacing along each nonvacuum axis. Dynamical matrices were interpolated from these grids onto paths between high-symmetry Brillouin zone points, as generated by the SeeK-path utility.^119,120^

Local mode analysis (LMA) and characterization of normal modes (CNM) were performed as described in the main text on the dynamical matrices obtained from CASTEP according to the equations presented in the Theoretical Background. These computations were performed in an in-house software implementing all methods discussed in this paper, which will be publicly released as an open-source Python package in the future.

## Results and Discussion

We here demonstrate the calculation and analysis of wave-vector-dependent behavior of local mode properties and normal mode characters. This is illustrated with increasingly complex systems, beginning with a one-dimensional polymeric system, followed by a two-dimensional material and finally extending the analysis to three-dimensional crystalline solids. We note that the aim of this discussion is not to uncover new physics in the models we present. Instead, our aim is to demonstrate the possibilities that are enabled by our extension of local vibrational mode theory and illustrate how local vibrational mode interpretations can be used to rationalize phonon dispersion behavior in terms of intuitive structural and chemical features.

### Wavevector-Dependent Local Mode Properties in a 1D system: Polyacetylene

To illustrate how local mode properties vary with wavevector, we first consider the local modes in two isomeric forms of a simple one-dimensional polymer: *cis*- and *trans*-polyacetylene (PAc), Figure 4. The periodic structure of each isomer consists of monomeric units repeating indefinitely along an arbitrary periodic axis (here, chosen to be the *z*-axis). Consequently, the Brillouin zone of each isomer is one-dimensional, with allowed wavevectors ranging from Γ = 0 to *Z* = π/*a*, where *a* is the length of the repeating unit. When **k** = Γ, atoms in all repeating units vibrate in-phase. In contrast, when **k** = *Z*, atoms in the neighboring repeating units vibrate with opposite phases. These phase relations modify the restoring forces experienced by the vibrating atoms at different wavevectors, leading to the variation of vibrational frequencies with respect to **k**, referred to as phonon dispersion, in Figure 5a,b.

**Figure 4:**
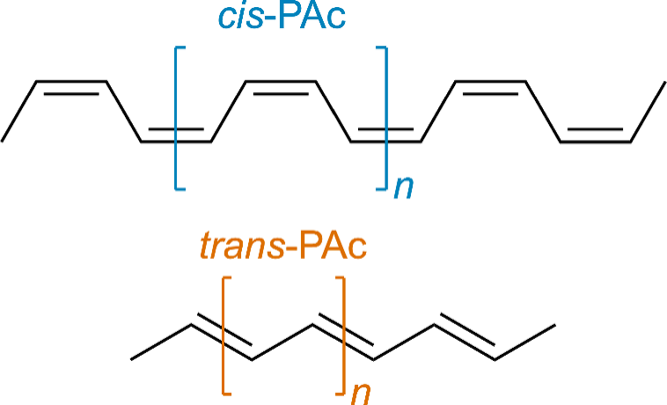
Periodic structures of the *cis*- and *trans*-isomers of polyacetylene (PAc). A portion of the one-dimensional chain is shown for each, with the repeating unit indicated in square brackets.

**Figure 5:**
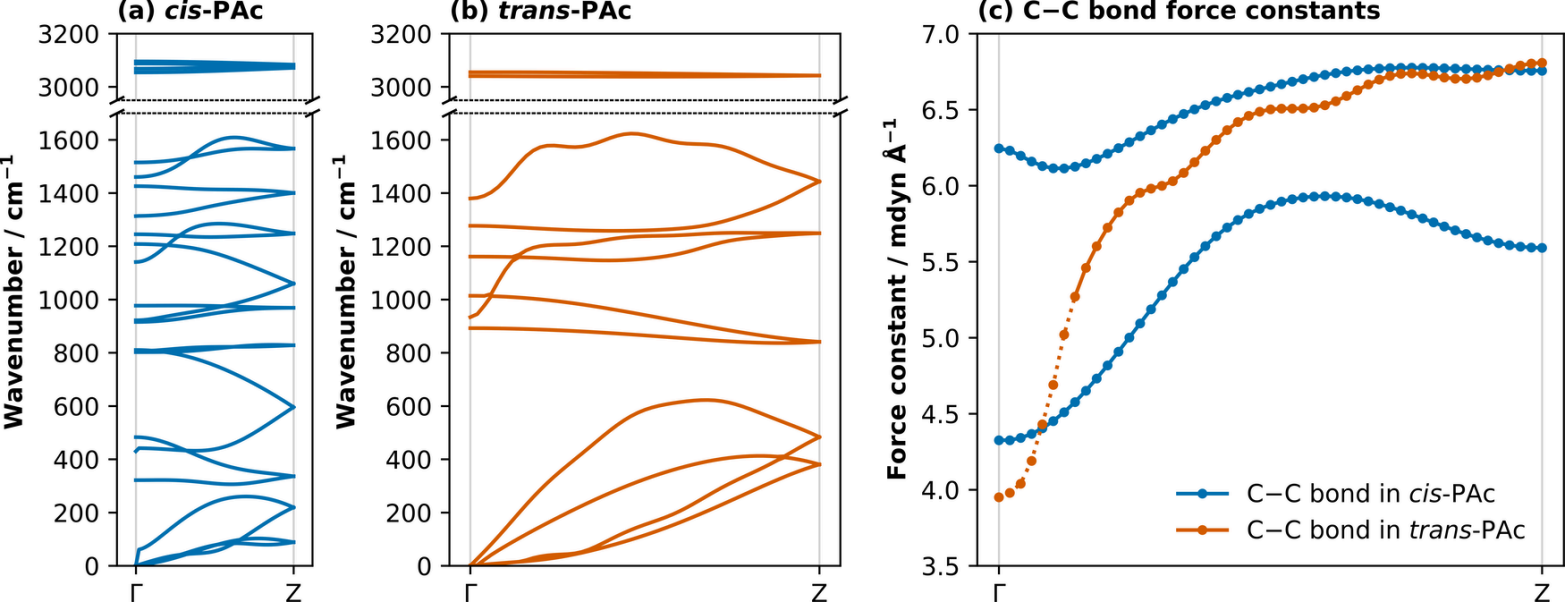
Local mode analysis of 1-dimensional polymeric systems. (a) Phonon dispersion in *cis*-PAc; (b) Phonon dispersion in *trans*-PAc; (c) Wavevector-dependence of local force constants for the C–C bonds in *cis*- and *trans*-PAc. In both (a) and (b), the scale of the *x*-axis represents the relative dimensions of the Brillouin zone of the two isomers, noting that the length of the repeat unit of *cis*-PAc is approximately twice that of *trans*-PAc. In (c), the scale of the *x*-axis is normalized to illustrate how the local force constant is affected as neighboring repeating units transition from vibrating in-phase at Γ to out-of-phase at *Z*. The degeneracy of C–C bonds in *cis*-PAc is a result of Peierls distortion. The dotted portion in (c) represents local force constants interpolated across the near-Γ region of the phonon dispersion in *trans*-PAc, where numerical noise led to small imaginary frequencies for the near-zero phonon branches.

To probe how these phase-dependent interactions manifest in local vibrational properties, we performed a wave-vector-resolved LMA for both isomers of PAc. We computed the local force constants, eq 8, for the local vibrational modes along the C–C bond internal coordinates at a range of wavevectors between Γ and *Z*, and investigated their variation across the Brillouin zone, Figure 5c.

The simple example of PAc isomers demonstrates several key features of how local vibrational mode behavior changes with wavevector. First, analogous to the behavior of the phonon frequencies, the local mode force constants exhibit dispersion as a function of **k**. This is expected: for any given **k**, each local mode is a linear combination of the normal modes at that same **k**. Consequently, as normal mode behavior varies with **k**, so too do the local mode force constants. For example, the dispersion of the C–C bond local mode force constant in *trans*-PAc, Figure 5c, resembles the combined behavior of the phonon branches between ∼1000 and 1600 cm^–1^.

It follows that local mode force constants at **k** = Γ are unlikely to give an accurate representation of the stiffness of the internal coordinates in periodic systems. We expect this limitation to be most significant where the internal coordinate participates in strongly dispersive normal mode branches.

The **k**-dependence of local mode force constants offers the opportunity to probe long-range cooperative bonding behavior in periodic systems. In both isomers of PAc, the C–C bond force constant increases as **k** → *Z*. This increase indicates that the effective stiffness of the C–C bond internal coordinate is strongly dependent on the relative distortions of neighboring C–C bonds. We can interpret this **k**-dependence in PAc as reflecting the impact of internal-coordinate distortions on π-conjugation along the polymer chain. Out-of-phase distortions of C–C bond periodic images at **k** = *Z* disrupt the π-conjugation. In contrast, when all C–C bonds distort in-phase (at **k** = Γ), the π-conjugation is preserved (see further discussion in the ESI, S5). This is to say, bonding in periodic polymers is correlated along the polymer chain.

The small change in molecular structure between *cis*-PAc and *trans*-PAc gives rise to disparate phonon dispersion behavior, as shown in Figures 5a and 5b. Using this example, we observe how LMA allows us to determine which chemical features give rise to the dispersion behavior. For instance, the C–C bonds in *cis*-PAc are, on average, stiffer than in *trans*-PAc, but are less strongly correlated between neighboring unit cells (i.e., they have weaker **k**-dependence). We believe that this weaker **k**-dependence relates to the smaller repeat unit length in *trans*-PAc, and acts to demonstrate the potential of our method to rationalize phonon dispersion behavior in terms of chemical features. In addition, our results highlight that, while wavevector-resolved local modes do not directly probe intrinsic bond strengths, the local mode force constants are indicative of how cooperative distortion patterns impact local chemical environments.

### Wavevector-Dependence of Local-Mode Characters in a 2D System: Graphene

Using a two-dimensional system as an example, we next explore how the local-mode contributions to the normal modes evolve across the Brillouin zone, leveraging the CNM framework, eq 11. Extending our investigation from one- to two-dimensions also allows us to study how normal mode characters change as a function of the direction of phonon propagation in reciprocal space. In doing so, we highlight the role that directional anisotropy in the Brillouin zone has in shaping the coupling between local and collective vibrational dynamics.

As a prototypical two-dimensional material, we elected to study graphene, a hexagonal lattice of carbon atoms with a two-atom unit cell, as shown in Figure 6a. Its Brillouin zone is likewise hexagonal, featuring high-symmetry points labeled as Γ (**k** = (0 0)), *K* ( $k=\left(\frac{1}{3}\frac{1}{3}\right)$ ), and *M* ( $k=\left(\frac{1}{2}0\right)$ ), defined in fractional coordinates, Figure 6b. Hence, graphene provides an ideal platform for sampling phase relations along different high-symmetry reciprocal space paths.

**Figure 6:**
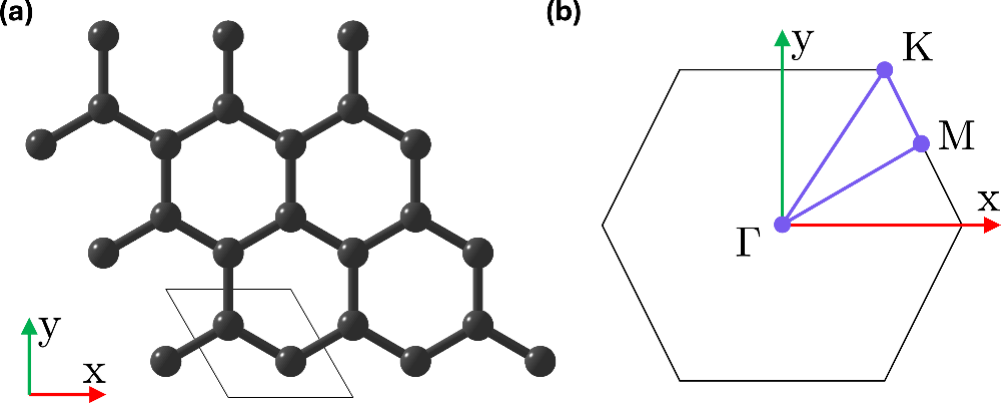
Crystal structure of monolayer graphene. (a) Periodic structure of graphene, showing the unit cell as black wires. (b) Brillouin zone of graphene, showing the key high-symmetry points.

To quantify the **k**-dependence local-mode contributions to each normal mode, we compute the similarity metric *C*_*n*,μ_, eq 11, between the graphene normal modes and a set of linearly independent local modes. These local modes were computed from bond internal coordinates along the two linearly independent nearest-neighbor bonding directions in the hexagonal lattice: *ŷ* and $\frac{\sqrt{3}}{2}\mathrm{x̂}-\frac{1}{2}ŷ$ . We define an additional internal coordinate that corresponds to the out-of-plane buckling of the hexagonal network, which is defined as the opposing motion of the two atoms in the unit cell along *ẑ*. An additional three orthogonal, basis-completing translational coordinates are included in our analysis to ensure we consider a complete representation of the vibrational space, Figure 7a (see Interpreting Local Modes in Periodic Systems for discussion). To analyze our calculations, we identify the local mode that has the largest contribution to each phonon branch across the Brillouin zone, Figure 7b. We additionally perform at each k-point a full decomposition of the normal modes into the contributions from the full set of local modes, Figure 7c.

**Figure 7:**
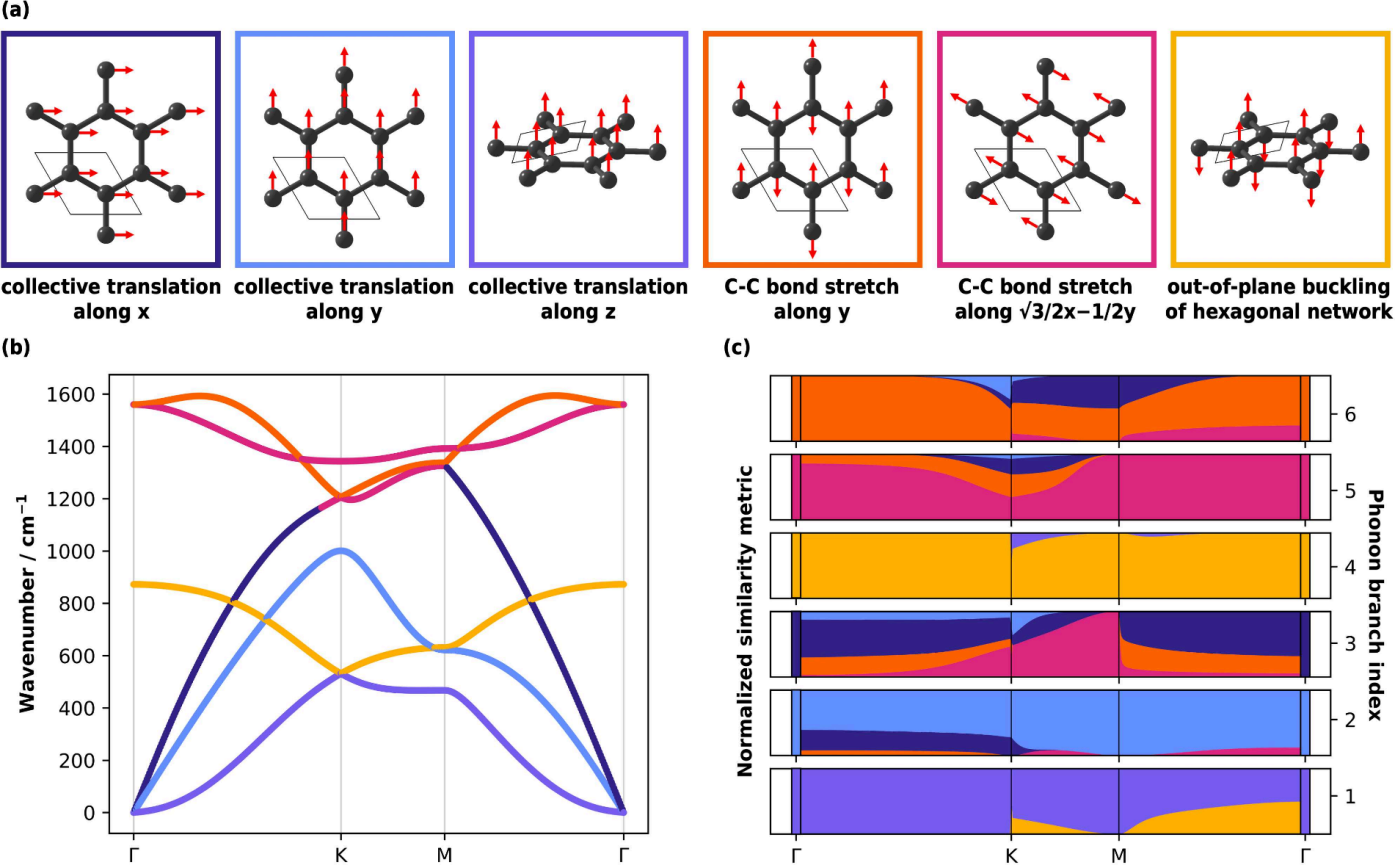
Phonon and local mode behavior of monolayer graphene. (a) Γ-point vibration patterns for the local modes defined along a set of linearly independent internal coordinates and special translational coordinates selected for LMA of graphene. The red arrows represent the direction of atomic motion. (b) Phonon dispersion relations of graphene along high-symmetry paths in the Brillouin zone (Γ → *K* → *M* → Γ), colored according to the local mode with the largest fractional similarity metric *C*_*n*,μ_ at each k-point. (c) **k**-Dependence of the fractional similarity metric *C*_*n*,μ_, showing the relative similarity between each normal mode and the set of local modes (a), at each k-point. The boxes drawn at each end of the plot represent *C*_*n*,μ_ computed exactly at the Γ-point, with clear lifting of mode degeneracies at finite wavevectors. For (c), branch connectivity was determined by ensuring the smooth evolution of *C*_*n*,μ_ across the Brillouin zone.

The local-mode character evolves continuously along each phonon band when traced along a continuous high-symmetry path, Figure 7c. Along the path Γ → *K* the similarity metric *C*_*n*,μ_ shows that the first acoustic band corresponds to the collective *z*-axis translation of graphene (purple), but that along the *M* → Γ path this same mode can be partially described by the out-of-plane buckling coordinate (yellow). In fact, we see that this acoustic normal mode becomes increasingly similar to the out-of-plane puckering coordinate (yellow) as we approach Γ from the *M*-direction. This is a particularly interesting feature, as it highlights how the behavior of collective motions in crystals is intimately dependent on the path being considered through reciprocal space. Similarly, we see for the highest frequency optical branch (branch 6) that, while the normal mode corresponds entirely to the *y*-axis C–C bond stretch (orange) across the majority of the Γ → *K* path, its behavior becomes similar to the *x*- and *y*-axis translational coordinates (light and dark blue) as it approaches *K* and the normal-mode frequency softens. Hence, by following the evolution of the CNM metric across the Brillouin zone, we can directly observe how the character of vibrational dynamics evolves and its relation to the structure of the phonon dispersion relation.

While the local-mode character of normal modes evolves continuously along continuous segments in the Brillouin zone, discontinuities are observed as we change the direction. At junctions between distinct high-symmetry directions, symmetry-enforced degeneracies permit a redistribution of local-mode character among neighboring phonon branches. This preserves certain components of the local-mode character, but allows others to be exchanged between degenerate modes (e.g., see branches 1, 4 and 3, 6 at *K* and *M*). This behavior provides a local-mode-resolved description of phonon mode degeneracy, which seems to distinguish true branch crossings, where no character mixing occurs and the two modes are distinct by symmetry, from avoided crossings, where modes of compatible symmetry couple through shared local distortions. This information is inaccessible from the phonon dispersion alone and is difficult to extract from normal mode eigenvectors owing to their nonuniqueness within degenerate subspaces. Hence we anticipate that our LMA approach will enable chemically intuitive rationalization of branch-specific contributions to the physical behavior of periodic solids.

It follows from the above that wavevector-resolved CNM provides a route to determine whether phonon band dispersion originates from a change in the stiffness of a particular displacement pattern under in-phase or out-of-phase vibrations or a change in the displacement pattern altogether. The former is the case for the branch at ∼500–900 cm^–1^ (branch 4 in [Fig fig7]c), whose character most strongly matches the out-of-plane buckling internal coordinate across the Brillouin zone. Hence, for branch 4, the dispersion behavior arises from how periodic images of the same interatomic interaction influence one another across the lattice. This appears to be similar for the other branches (branches 4–6) that correspond to intramolecular distortions. In contrast, for branch 3 (an external vibrational mode), the phase relationships give rise to completely different distortion patterns across the Brillouin zone. We anticipate that such information will be useful for identifying how to fine-tune the phonon dispersion behavior.

### Wavevector-Resolved LMA and CNM in 3D Systems: Magnesium Oxide and Potassium Magnesium Fluoride

Having shown the capabilities of wavevector-resolved LMA and CNM based on the simple 1D and 2D model systems PAc and graphene, we now demonstrate the applications of our approach to three-dimensional systems. For this demonstration, we pick magnesium oxide, MgO, and potassium magnesium fluoride, KMgF_3_, and we illustrate how visualizing local-mode characters atop phonon dispersion plots can aid in the interpretation of the vibrational dynamics of crystals.

#### Local-Mode Characters at a Glance in Magnesium Oxide, MgO

MgO crystallizes in the rock-salt structure (*Fm*3̅*m* space group) and has a simple two-atom unit cell, Figure 8a. The vibrational space of this crystal can be spanned by a set of local modes constructed along three perpendicular Mg–O bond internal coordinates, and three basis-completing ‘special’ translational coordinates along *x̂*, *ŷ* and *ẑ*. We note that the Γ-point B-matrix constructed from this internal coordinate set has mutually orthogonal rows, leading to a fully orthogonal set of local modes at Γ; this is a convenient reference, but the same analysis can be performed with arbitrary sets of internal coordinates. Using this set of internal coordinates, we performed **k**-resolved LMA and CNM for MgO. We demonstrate that with our approach it becomes possible to represent how different chemical moieties contribute to the phonon dispersion behavior in a standard dispersion plot. To achieve this, we color the phonon branches across a dense sampling of wavevectors on a high-symmetry Brillouin zone path according to the cumulative *C*_*n*,μ_ computed for all Mg–O bond internal coordinates. The resulting plot, Figure 8b, illustrates which portions of the phonon spectrum correspond to vibrations that resemble Mg–O bond stretching. We note that, because our framework is a direct analysis of the dynamical matrix, any nonanalytic corrections that arise in the vibrational dynamics of solids, such as LO–TO splitting in polar systems (e.g., MgO), are inherently accounted for in our analysis.

**Figure 8:**
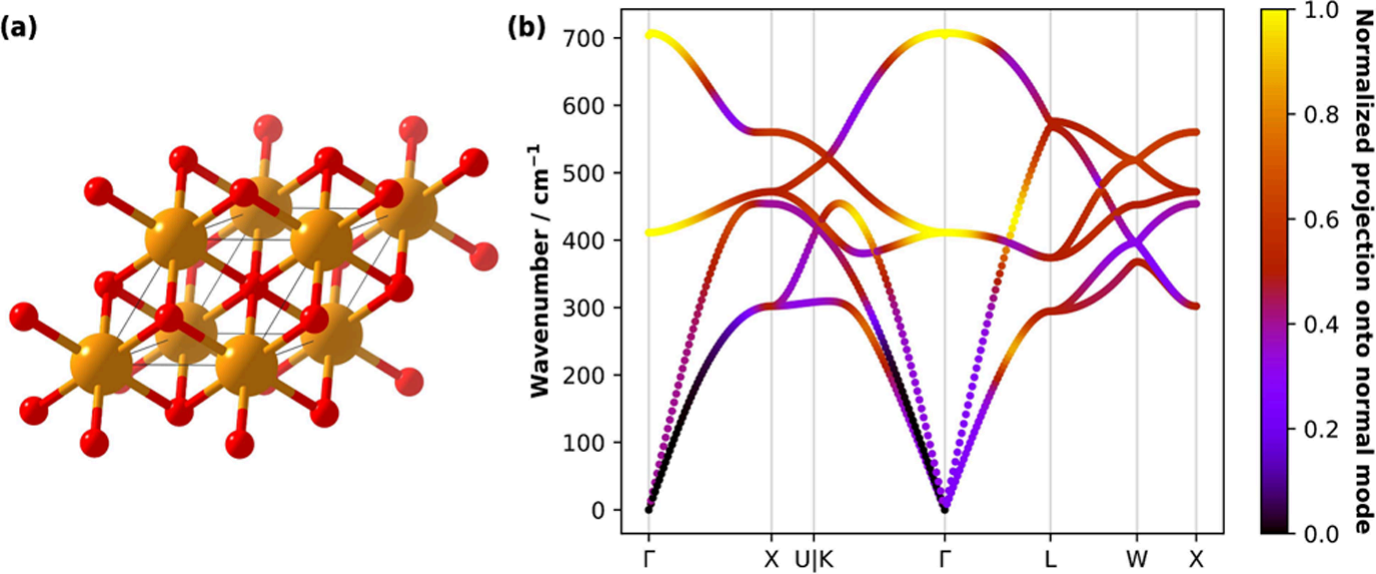
LMA of MgO. (a) Crystal structure of MgO (space group *Fm*3̅*m*), showing atoms (Mg: orange; O: red) in and around the primitive cell (shown as black wires). (b) Phonon dispersion curves in MgO colored by the relative magnitude of the Mg–O bond stretching character of the phonon branches across high-symmetry points in the Brillouin zone.

This approach provides an immediate visual measure of how strongly a given phonon branch resembles distortions along a selected internal coordinate, giving access to a chemically intuitive real-space interpretation of phonon dispersion behavior to complement the formal description provided by the phonon eigenvectors. We anticipate that this work will have practical implications for a range of problems in materials science. By identifying which portions of the phonon dispersion curve involve distortions of specific bonds or functional groups, the method facilitates the chemically informed analysis of properties such as lattice thermal transport, mechanical response, and vibrationally driven reactivity. For instance, its application to mechanically responsive and energetic crystals would enable direct identification of phonon branches associated with distortions of reactive moieties, while in extended inorganic solids, it could help elucidate how phase-dependent bond distortions contribute to branch dispersion and stiffness. More generally, this local-mode-based coloring provides a transferable framework for interpreting phonon spectra in terms of real-space dynamics.

#### Large Internal Coordinate Sets in Potassium Magnesium Fluoride, KMgF_3_

We have so far demonstrated the use of our method on simple systems with small sets of internal coordinates. As our final example, we study the perovskite KMgF_3_, whose five-atom unit cell, Figure 9a, necessitates a set of 15 linearly independent internal coordinates to define local modes that span the vibrational space of this system. A graph-theoretic search followed by the removal of redundant internal coordinates yielded three Mg–F bonds, six K–F interactions (defined as K–F internuclear distances), two 90° F–Mg–F angles and one ca. 215° Mg–F–K–F dihedral, complemented by three “special” translational coordinates, see ESI, S1 and S4.4. We perform wavevector-resolved LMA and CNM for KMgF_3_ using this set of internal coordinates, and plot the phonon dispersion relation for this system, with bands colored by which type of internal coordinate has the maximum cumulative *C*_*n*,μ_ contribution at each k-point, Figure 9b.

**Figure 9:**
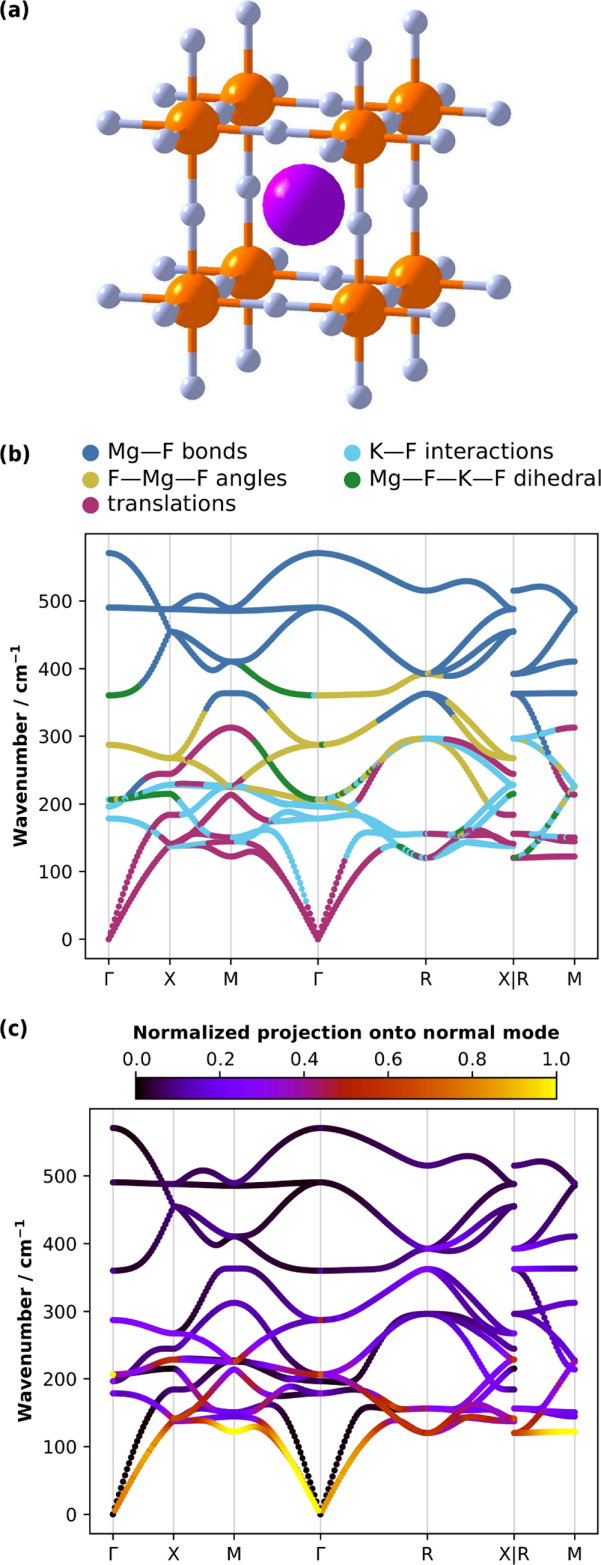
CNM in KMgF_3_. (a) Crystal structure of KMgF_3_ (space group *Pm*3̅*m*) showing atoms (K: purple, Mg: orange, F: blue) in and around the primitive cell. The boundaries of the cubic unit cell are obscured by Mg–F bonds. (b) Phonon dispersion in KMgF_3_ colored according to which local mode type most strongly contributes to the character of the phonon branches, plotted along high-symmetry paths in the Brillouin zone. (c) Phonon dispersion colored according to the relative contributions of the MgF_6_ octahedra tilting local modes to the phonon branches.

Mapping the contributions from all internal coordinates at once yields an overview of the vibrational behavior of the studied system, which would otherwise require a laborious inspection of the phonon eigenvectors. The phonon spectrum clearly consists of frequency regions where specific types of distortions dominate, e.g., Mg–F bond stretches dominate the 400–600 cm^–1^ region, and K–F interactions dominate the 150–250 cm^–1^ region. Moreover, as shown for graphene, we can infer branch connectivity by tracking the local-mode characters across the high-symmetry Brillouin zone paths. We note that, in certain regions of Figure 9b, branch characters appear to rapidly switch between different internal coordinates. This behavior is observed along high-symmetry paths between special points in the Brillouin zone in the vicinity of mode degeneracies. In these regions, local-mode contributions are allowed to redistribute continuously between neighboring branches; hence, this behavior is intrinsic to the local-mode representation of vibrational dynamics and does not affect the underlying phonon continuity.

In perovskite materials, low-energy distortions involving cooperative tilting of the corner-sharing MgF_6_ octahedra play key roles in both structural phase transitions and lattice instabilities. Our method can directly probe these structurally important distortions by treating local modes defined by arbitrary internal coordinates. For KMgF_3_ we define a set of local modes that describe rigid-body rotations (tilts) of the MgF_6_ octahedron around its center of mass, ESI, S4.4. To quantify the contributions of these rigid-body local modes to the phonon dispersion, we compute the relative overlap amplitudes, *A*_*n*,μ_ (eq 10), and color the phonon branches accordingly, Figure 9c. This analysis shows that the octahedral tilting character is concentrated in the low-frequency region of the phonon spectrum, where it contributes strongly to acoustic and low-lying optical branches. Hence, our method captures the physics of these rigid-body modes, which involve collective weakly restoring distortions of the lattice framework rather than localized bond stretching. The ability to isolate such tilting contributions across the Brillouin zone illustrates how our wavevector-resolved local mode framework enables the direct interpretation of specific phonon dispersion features in terms of chemically and structurally meaningful collective distortions. While such collective coordinates go beyond simple bond, angle, or dihedral definitions, they are fully compatible with our formalism and can be incorporated, provided their derivatives are well-defined.

Of course, further insight into the relative similarity metrics between the local modes and each normal mode at a given k-point can be obtained via the conventional way of displaying local-mode characters, where the *C*_*n*,μ_ values are shown as bar charts for the full set of normal modes. We present this analysis for the high-symmetry points in the Brillouin zone of KMgF_3_, as shown in Figure 10.

**Figure 10:**
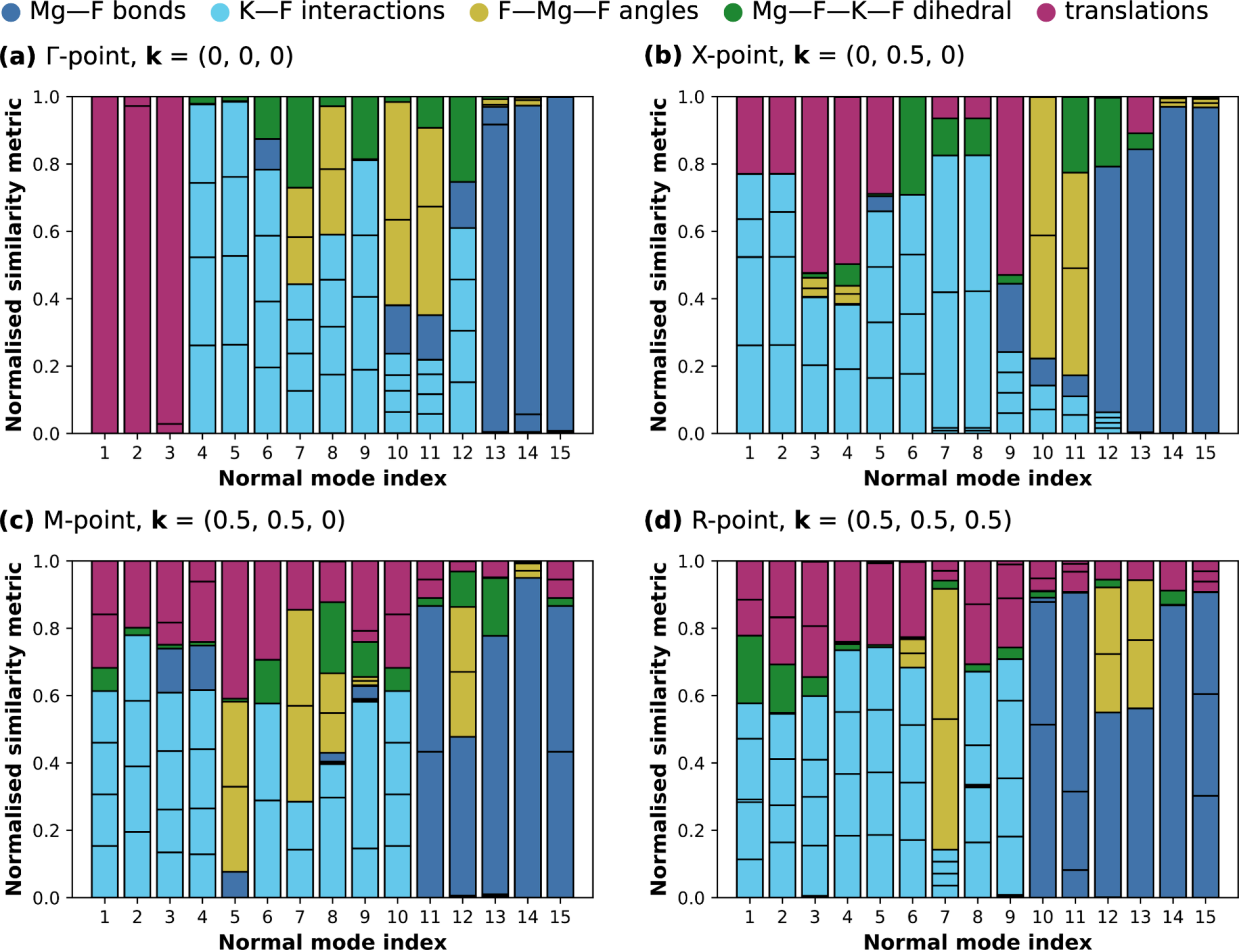
Decomposition of normal modes at the high-symmetry points in the Brillouin zone of KMgF_3_ in terms of the normalized similarity metric, *C*_*n*,μ_, computed for local modes constructed from a set of linearly independent internal coordinates. (a) Γ-point, **k** = (0, 0, 0); (b) *X*-point, **k** = (0, 0.5, 0); (c) *M*-point, **k** = (0.5, 0.5, 0); (d) *R*-point, **k** = (0.5, 0.5, 0.5).

## Conclusions

This work presents a generalized local vibrational mode framework that enables wave vector-resolved characterization of vibrational dynamics in periodic systems. By extending the local mode analysis (LMA) and characterization of normal modes (CNM) formalisms, we demonstrate how chemically intuitive, internal-coordinate-based descriptors can be used to rationalize phonon dispersion behavior in one-, two-, and three-dimensional periodic materials. In particular, we demonstrate a number of unique analyses that are made possible using our new methodology.

We first show using a 1D polymeric system that local mode force constants can be calculated for specific chemical moieties as a function of the wavevector. Using these wavevector-dependent local mode force constants, it becomes possible to explore long-range cooperative bonding in periodic systems and interpret the chemical origins of phonon dispersion relations.

By subsequently using the CNM approach to study a 2D system, graphene, we demonstrate the ability to quantify how normal mode characters evolve across the Brillouin zone. We highlight how our method clearly shows that the evolution of normal mode character is continuous but depends on the direction of the chosen path through the Brillouin zone. This behavior makes our tools extremely useful for qualifying branch connectivity across phonon dispersion relations.

Finally, for a set of representative 3D systems, we highlight how complete phonon dispersion relations can be interpreted and visualized in terms of local vibrational mode character. This not only rationalizes the wavevector-dependence of phonon frequencies in terms of chemical structure but also enables predictive interpretations of how structural modifications might alter dispersion characteristics. Hence, our analysis opens new opportunities to ‘engineer’ phonon dispersion relations with chemically informed precision.

In its current form, our method is an analysis of harmonic normal modes and is therefore subject to the limitations of the harmonic approximation. Consequently, if wavevector-resolved force constants are used as bond strength indicators, care must be taken when analyzing strongly anharmonic systems. Furthermore, the specific choice of the internal coordinate set strongly influences the interpretation of CNM amplitudes and hence any derived chemical insights. With these considerations in mind, our method nevertheless provides a robust framework to characterize the vibrational behavior in periodic materials in terms of local structural motifs. While we have demonstrated the application of our method to only small periodic systems, the analysis can be readily performed on systems with hundreds of atoms on a personal computer within seconds. Correspondingly, we do not expect any significant limitations for the expansion of our method to analyze much larger periodic systems.

We anticipate that our wavevector-resolved local mode framework will serve as a versatile tool for the community, enabling chemically intuitive analyses of phonon behavior and guiding the design of materials with tailored vibrational and functional properties.

## Supplementary Material



## Data Availability

Raw data are available in the Zenodo repository at: 10.5281/zenodo.18302794. The code implementing the methods developed in this work will be made available open source on the CATCO GitHub repository, https://github.com/SMU-CATCO.

## Notes

Further simulation details and analyses (PDF)
