# Supplementary material for: Local Vibrational Mode Analysis of Phonon Dispersion Relations in Crystals
Source: J Chem Theory Comput. 2026 Feb 26;22(9):4742–57. doi: 10.1021/acs.jctc.6c00097 (PMC13173525; doi:10.1021/acs.jctc.6c00097)
Supplement: Supplementary file 1 [file ct6c00097_si_001.pdf]

SUPPLEMENTARY INFORMATION

Local vibrational mode analysis of phonon dispersion relations in crystals

Mateusz Mojsak,[†] Filippo Bodo,[‡,¶] Alessandro Erba,[‡] Adam A. L. Michalchuk,[*,†,§] and Elfi Kraka[*,¶]

†*School of Chemistry, University of Birmingham, Birmingham, UK, B15 2TT*

‡*Dipartimento di Chimica, Università di Torino, via Giuria 5, 10125 Torino, Italy*

¶*Department of Chemistry, Southern Methodist University, 3215 Daniel Ave, Dallas, Texas, 75275-0314, USA*

§*Federal Institute for Materials Research and Testing (BAM), Richard-Wilstaetter-Str 11, 12489, Berlin, Germany*

E-mail: a.a.l.michalchuk@bham.ac.uk; ekraka@smu.edu

Contents

S1 Graph-theoretic internal coordinate search algorithm **3**

S2 Equivalence between methods for calculating the B matrix in periodic systems **4**

S2.1 Mathematical equivalence 4

S2.2 Numerical equivalence 6

S3 Regularization of zero and near-zero eigenvalues **7**

S4 Model systems **11**

S4.1 *cis*- and *trans*-Polyacetylene 11

S4.2 Graphene 13

S4.3 MgO Rock Salt 15

S4.4 $KMgF_3$ Perovskite 16

S5 Bond order analysis under local mode distortions in *cis*- and *trans*-polyacetylene **19**

References **21**

S1 Graph-theoretic internal coordinate search algorithm

A method for the automatic definition of bond, angle, and dihedral internal coordinates in molecular systems has previously been described by Moura et al..[1] Our automatic internal coordinate search is based on the same graph-theoretic algorithm, adapted here to periodic systems by operating on a cluster constructed around the primitive cell and filtering redundant periodic images of equivalent internal coordinates, as described in the main text. Below, we summarize the key steps of the algorithm while noting the choices of reference atoms to ensure that unique representations in the primitive cell are selected.

1. A bond graph is constructed from the atomic coordinates in the cluster by applying element-pair-specific distance cutoffs, yielding a binary connectivity matrix that encodes all bonded interactions.

2. **Bonds** are identified by enumerating all unique *two-atom* chains (bonded pairs) in the connectivity graph. Only bonds for which the *first* atom in the canonical representation (i.e., a unique ordering of atom indices used to avoid duplicates) belongs to the reference unit cell are retained, ensuring a unique representation of each bond.

3. **Angles** are identified by enumerating all unique *three-atom* chains A–B–C in the connectivity graph, where atoms A and C are bonded to a common central atom B. Only angles with the central atom B located in the reference unit cell are retained, ensuring uniqueness.

4. **Dihedrals** are identified by enumerating all unique *four-atom* chains A–B–C–D in the connectivity graph, where A–B, B–C, and C–D are bonded. The *second* atom in the canonical representation, B, is required to lie in the reference unit cell. Reverse duplicates and translationally equivalent dihedrals arising from periodic images are identified and removed by explicit comparison of translated geometries.

In addition to bond, angle and dihedral internal coordinates, this procedure allows any

coordinates that can be formally described based on the bond graph (e.g., higher-order chain or ring coordinates) to be automatically searched for to generate all unique representations.

S2 Equivalence between methods for calculating the B matrix in periodic systems

In the main text, we introduce two methods to account for the wavevector-dependent atomic displacement patterns within our periodic local mode analysis (LMA) formalism: the *Expanded B-Matrix Method*, and the *k-Dependent B-Matrix Method*. Both methods ensure that, at the point of constructing the $\mathbf{D}(\mathbf{k})$ matrix for any particular wavevector, we correctly handle the phase relationships between atoms in the cluster around the primitive cell. For internal coordinates that are fully contained within a single unit cell, our proposed equations reduce to the standard formalism used for LMA in isolated molecules. Here, we demonstrate the mathematical equivalence between the *Expanded B-Matrix Method* and the *k-Dependent B-Matrix Method*, and provide representative $\mathbf{D}(\mathbf{k})$ and $\mathbf{G}$ matrices to illustrate this equivalence.

S2.1 Mathematical equivalence

The *Expanded B-Matrix Method* relies on expanding the $\mathbf{B}$ and $\mathbf{L}(\mathbf{k})$ matrices. This expansion allows us to include elements that correspond to all of the atoms that are contained in the cluster being used to define the desired set of internal coordinates. The elements of the $\mathbf{D}$ matrix at wavevector $\mathbf{k}$ are given by

$$D_{n,\mu}(\mathbf{k}) = \mathbf{b}^*_{n\cdot} \cdot \mathbf{l}^*_{\cdot\mu}(\mathbf{k}) = \sum_{\lambda\kappa\alpha} \left(\frac{\partial q_n}{\partial x_{\lambda i\alpha}} l_{\kappa\alpha,\mu}(\mathbf{k}) e^{i\mathbf{k}\cdot\mathbf{R}_\lambda} \right). \tag{S2.1}$$

In Eq. S2.1, the rows of $\mathbf{B}$, $\mathbf{b}^*_{n\cdot}$, have been expanded to store internal coordinate derivatives associated with atoms outside the reference unit cell. Similarly, the columns of $\mathbf{L}(\mathbf{k})$, $\mathbf{l}^*_{\cdot\mu}(\mathbf{k})$, have been also expanded to describe the displacement pattern associated with all of the

atoms in the cluster, done by leveraging the relationship

$$u_{\lambda\kappa\alpha,\mu}(\mathbf{R}) = l_{\kappa\alpha,\mu}(\mathbf{k})\, e^{i\mathbf{k}\cdot\mathbf{R}_\lambda}. \tag{S2.2}$$

In Eq. S2.1, we highlight the terms that are dependent on the unit cell index λ in **red**, and terms that are independent of the unit cell in **blue**. Since the terms $l_{\kappa\alpha,\mu}(\mathbf{k})$ are independent of the unit cell index λ (*i.e.,* only the Bloch phase factor in $\mathbf{l}^*_{\cdot\mu}(\mathbf{k})$ varies between cells), they can be factored out of the sum over λ, such that

$$D_{n,\mu}(\mathbf{k}) = \sum_{\kappa\alpha}\Bigg[\underbrace{\sum_{\lambda}\left(\frac{\partial q_n}{\partial x_{\lambda i\alpha}} e^{i\mathbf{k}\cdot\mathbf{R}_\lambda}\right)}_{B_{n,i\alpha}(\mathbf{k})} \times l_{\kappa\alpha,\mu}(\mathbf{k})\Bigg]. \tag{S2.3}$$

In so doing, we can effectively re-define the $\mathbf{B}$ matrix such that the derivatives with respect to all periodic images of each atom in the cluster are summed into the corresponding element in the reference cell, multiplied by the corresponding Bloch phase factor. This removes the need to expand the $\mathbf{B}$ and $\mathbf{L}(\mathbf{k})$ matrices, and results in a $\mathbf{k}$-dependent $\mathbf{B}(\mathbf{k})$ matrix that respects periodic boundary conditions,

$$B_{n,i\alpha}(\mathbf{k}) = \sum_{\lambda} \frac{\partial q_n}{\partial x_{\lambda i\alpha}} e^{i\mathbf{k}\cdot\mathbf{R}_\lambda}, \tag{S2.4}$$

while yielding an identical definition for the elements of the $\mathbf{D}(\mathbf{k})$ matrix.

Importantly, the inclusion of phase factors in $\mathbf{B}(\mathbf{k})$ does not alter the diagonal (real) elements of the $\mathbf{G}$ matrix (where $\mathbf{G} = \mathbf{B}\mathbf{M}^{-1}\mathbf{B}^\dagger$) as compared with the *Expanded B-Matrix Method*. The only difference is that, in the *k-Dependent Method*, the off-diagonal elements are nonzero and arise from the combination of Bloch phases across different unit cells. In the context of LMA, only the diagonal elements are needed, as they are used to determine local mode frequencies; the off-diagonal elements do not enter the calculation.

S2.2 Numerical equivalence

Here, we demonstrate the numerical equivalence of the *Expanded B-Matrix* and *k-Dependent B-Matrix* methods. As a representative example, we perform our analysis as described in the main text on the dynamical matrix computed for a low-symmetry k-point, $\mathbf{k} = (0.500000\ 0.268293\ 0.731707)$, in the model rock-salt system, MgO. The choice of a low-symmetry k-point is to ensure that the Bloch factors are non-trivial. Additionally, we highlight that all internal coordinates that we study in MgO involve atoms that span multiple unit cells.

D(k) matrix and diagonal G matrix elements calculated using the expanded B-matrix method

$$\mathrm{Re}(\mathbf{D}(\mathbf{k})) = \begin{pmatrix} -0.03271 & 0.08796 & 0.08359 & 0.03940 & 0.20856 & -0.20558 \\ 0.03271 & 0.08796 & -0.08359 & 0.03940 & -0.20856 & -0.20558 \\ -0.00000 & -0.02060 & 0.00000 & 0.24618 & -0.00000 & 0.03837 \\ -0.01870 & 0.04854 & 0.04367 & -0.02221 & -0.17028 & 0.16853 \\ 0.01870 & 0.04854 & -0.04367 & -0.02221 & 0.17028 & 0.16853 \\ -0.00000 & -0.02060 & -0.00000 & 0.24618 & -0.00000 & 0.03837 \end{pmatrix}$$

$$\mathrm{Im}(\mathbf{D}(\mathbf{k})) = \begin{pmatrix} 0.00593 & -0.01576 & -0.01469 & -0.00199 & -0.00442 & 0.00428 \\ 0.00593 & 0.01576 & -0.01469 & 0.00199 & -0.00442 & -0.00428 \\ -0.18796 & -0.00000 & -0.07626 & 0.00000 & 0.00109 & -0.00000 \\ -0.05175 & 0.13741 & 0.12810 & 0.01731 & 0.03853 & -0.03730 \\ -0.05175 & -0.13741 & 0.12810 & -0.01731 & 0.03853 & 0.03730 \\ 0.18796 & 0.00000 & 0.07626 & 0.00000 & -0.00109 & -0.00000 \end{pmatrix}$$

$$\mathrm{diag}(\mathbf{G}) = \begin{pmatrix} 0.10364614 & 0.10364614 & 0.10364614 & 0.10364614 & 0.10364614 & 0.10364614 \end{pmatrix}$$

D(k) matrix and diagonal G matrix elements calculated using the k-dependent B-matrix method

$$\mathrm{Re}(\mathbf{D}(\mathbf{k})) = \begin{pmatrix} -0.03271 & 0.08796 & 0.08359 & 0.03940 & 0.20856 & -0.20558 \\ 0.03271 & 0.08796 & -0.08359 & 0.03940 & -0.20856 & -0.20558 \\ -0.00000 & -0.02060 & 0.00000 & 0.24618 & -0.00000 & 0.03837 \\ -0.01870 & 0.04854 & 0.04367 & -0.02221 & -0.17028 & 0.16853 \\ 0.01870 & 0.04854 & -0.04367 & -0.02221 & 0.17028 & 0.16853 \\ -0.00000 & -0.02060 & -0.00000 & 0.24618 & -0.00000 & 0.03837 \end{pmatrix}$$

$$\mathrm{Im}(\mathbf{D}(\mathbf{k})) = \begin{pmatrix} 0.00593 & -0.01576 & -0.01469 & -0.00199 & -0.00442 & 0.00428 \\ 0.00593 & 0.01576 & -0.01469 & 0.00199 & -0.00442 & -0.00428 \\ -0.18796 & -0.00000 & -0.07626 & 0.00000 & 0.00109 & -0.00000 \\ 0.05175 & -0.13741 & -0.12810 & -0.01731 & -0.03853 & 0.03730 \\ 0.05175 & 0.13741 & -0.12810 & 0.01731 & -0.03853 & -0.03730 \\ -0.18796 & -0.00000 & -0.07626 & -0.00000 & 0.00109 & 0.00000 \end{pmatrix}$$

$$\mathrm{diag}(\mathbf{G}) = \begin{pmatrix} 0.10364614 & 0.10364614 & 0.10364614 & 0.10364614 & 0.10364614 & 0.10364614 \end{pmatrix}$$

Once the **G** and **D** matrices are obtained, the LMA and CNM calculations are performed identically for both methods. Hence, as these input matrices are numerical equivalents from both methods, it follows that both yield identical LMA and CNM results.

S3 Regularization of zero and near-zero eigenvalues

At the Γ-point, the presence of exact zero eigenvalues associated with rigid translations (and, in 1D systems, rotations) renders inverse operations in Eqs 7 and 8 in the main text formally undefined. This is not a numerical artifact but a fundamental consequence of translational invariance in harmonic vibrational theory, which is reached only in the strict limit of vanishing eigenvalues. To obtain well-defined local mode properties while retaining the complete normal-mode basis, we ensure matrix invertibility by regularizing these eigenvalues.

This is done by replacing the exact zeros with infinitesimal positive values, as described in the main text.

In practice, numerical phonon calculations rarely produce exact zeros, as is the case for all our model systems. Importantly, the regularization procedure does not alter the non-zero vibrational frequencies and eigenvectors, or any local mode property that corresponds to internal coordinates other than the special translational and rotational coordinates. As a representative example, we demonstrate the effects of regularization on LMA calculations performed using the Γ-point dynamical matrix computed for our model system, MgO.

To demonstrate that the regularization procedure has no effect on the nonzero phonon spectrum being studied, we diagonalized the dynamical matrix,

$$\mathbf{\Lambda} = \mathbf{L}^{\dagger}\mathbf{W}\mathbf{L} \tag{S3.1}$$

and regularized eigenvalues below a small threshold ε,

$$\widetilde{\mathbf{\Lambda}}_{ii} = \begin{cases} \mathbf{\Lambda}_{ii}, & \mathbf{\Lambda}_{ii} \geq \varepsilon, \\ \varepsilon, & \mathbf{\Lambda}_{ii} < \varepsilon. \end{cases} \tag{S3.2}$$

A regularized dynamical matrix was then reconstructed as

$$\widetilde{\mathbf{W}} = \mathbf{L}\widetilde{\mathbf{\Lambda}}\mathbf{L}^{\dagger}. \tag{S3.3}$$

Phonon frequencies were obtained from the eigenvalues $\widetilde{\lambda}$ according to

$$\omega_{\mu} = \mathrm{sign}(\widetilde{\lambda}_{\mu})\sqrt{|\widetilde{\lambda}_{\mu}|}, \tag{S3.4}$$

with appropriate unit conversion.

Notably, even for orders-of-magnitude different choices of ϵ,the non-zero frequencies computed for MgO remain unaffected, Table S3.1. Hence, we demonstrate that regularization

does not affect phonon frequencies corresponding to eigenvalues $> \varepsilon$. We also note that, by construction, this procedure does not affect the phonon eigenvectors.

To demonstrate that the regularization procedure does not impact the computed LMA results, we performed LMA using the Γ-point dynamical matrix for MgO as described in the main text, using internal coordinates given in Table S4.8. We present the computed local mode frequencies in Table S3.2. Regularization only affects the local mode frequencies for the special translational coordinates, designed to map onto the translational subspace of the normal mode basis that corresponds to the regularized eigenvalues at the Γ point. Additionally, replacing zero eigenvalues with infinitesimal positive values allows non-zero CNM similarity metrics between the acoustic normal modes and the local modes. However, we have verified that these contributions are negligible across the entire range of sampled ε values, only reaching $C_{n,\mu} \approx 10^{-3}$ when $\varepsilon = 10^{-4}$.

We emphasize that the regularization procedure is designed solely to stabilize the local mode formalism and preserve the physical vibrational spectrum; it must not be used to compensate for imaginary phonon frequencies arising from structural instabilities or incomplete geometry optimization.

Table S3.1: Phonon frequencies computed for different values of the parameter ε. The eigenvalues of the dynamical matrix are typically in the range 10^{-4}–10^{0} Hartree/bohr^2, and the value of ε used for regularization should be several orders of magnitude smaller to consider it 'infinitesimal'.

	Phonon frequencies / cm^{-1}					
Mode	without regularization	ε / Hartree bohr^{-2} 10^{-12}	10^{-10}	10^{-8}	10^{-6}	10^{-4}
1	−0.0314875	0.00514050	0.05140487	0.51404871	5.14048714	51.4048714
2	−0.0314875	0.00514050	0.05140487	0.51404871	5.14048714	51.4048714
3	0.83879410	0.83879410	0.83879410	0.83879410	5.14048714	51.4048714
4	411.268201	411.268201	411.268201	411.268201	411.268201	411.268201
5	411.268201	411.268201	411.268201	411.268201	411.268201	411.268201
6	703.557669	703.557669	703.557669	703.557669	703.557669	703.557669

Table S3.2: Local mode frequencies computed for different values of the parameter ε. Modes 1–3 correspond to bond stretching local modes, and modes 4–6 correspond to special translational coordinates, see Table S4.8.

Mode	Local mode frequencies / cm^{-1}					
	without regularization	ε / Hartree $bohr^{-2}$				
		10^{-12}	10^{-10}	10^{-8}	10^{-6}	10^{-4}
1	411.2669	411.2669	411.2669	411.2669	411.2669	411.2669
2	703.5574	703.5574	703.5574	703.5574	703.5574	703.5574
3	411.2669	411.2669	411.2669	411.2669	411.2669	411.2669
4	0.000000	0.0053	0.0525	0.5253	5.2532	52.5142
5	0.000000	0.0053	0.0525	0.5253	5.2532	52.5262
6	0.000000	0.0053	0.0525	0.5253	5.2532	52.5142

S4 Model systems

Here, we provide the input and optimized geometries for our model systems, alongside the phonon dispersion plots that represent the phonon spectra on which wavevector-resolved LMA and CNM were performed, and the internal coordinate sets that were used to construct the local modes. Dynamical matrices computed in CASTEP as described in the main text, as well as our complete LMA and CNM data including local mode force constants, frequencies, adiabatic vectors and CNM overlap matrices, are stored in an open-source Zenodo repository, in HDF5 format: https://doi.org/10.5281/zenodo.18302794.

S4.1 *cis*- and *trans*-Polyacetylene

Input and optimized geometry

Table S4.1: Input and optimized unit cell parameters and fractional atomic coordinates for ***cis*-polyacetylene**. The input geometry was generated manually. The 10 Å vacuum layers in the x- and y-directions ensure that the system is modeled as effectively one-dimensional.

	Input	Optimized
a (Å)	10.0000	10.0000
b (Å)	10.0000	10.0000
c (Å)	5.14965	4.45919
α (deg)	90	90
β (deg)	90	90
γ (deg)	90	90
Vol (Å^3)	514.9648	445.9193
Atom	$(a, b, c)_{\text{in}}$	$(a, b, c)_{\text{opt}}$
C 1	(0.45000, 0.50000, 0.12500)	(0.345306, 0.500000, 0.263376)
C 2	(0.55000, 0.50000, 0.37500)	(0.654694, 0.500000, 0.236624)
C 3	(0.55000, 0.50000, 0.62500)	(0.654694, 0.500000, 0.763376)
C 4	(0.45000, 0.50000, 0.87500)	(0.345306, 0.500000, 0.736624)
H 1	(0.35000, 0.50000, 0.17500)	(0.443302, 0.500000, 0.155716)
H 2	(0.65000, 0.50000, 0.32500)	(0.556698, 0.500000, 0.344284)
H 3	(0.65000, 0.50000, 0.67500)	(0.556698, 0.500000, 0.655716)
H 4	(0.35000, 0.50000, 0.82500)	(0.443302, 0.500000, 0.844284)

Table S4.2: Input and optimized unit cell parameters and fractional atomic coordinates for ***trans*-polyacetylene**. The input geometry was generated manually. The 10 Å vacuum layers in the x- and y-directions ensure that the system is modeled as effectively one-dimensional.

	Input	Optimized
a (Å)	10.0000	10.0000
b (Å)	10.0000	10.0000
c (Å)	2.38075	2.46843
α (deg)	90	90
β (deg)	90	90
γ (deg)	90	90
Vol (Å^3)	238.0746	246.8429
Atom	$(a, b, c)_{\mathrm{in}}$	$(a, b, c)_{\mathrm{opt}}$
H 1	(0.45000, 0.50000, 0.25000)	(0.35794, 0.500000, 0.250000)
H 2	(0.55000, 0.50000, 0.75000)	(0.64206, 0.500000, 0.750000)
C 1	(0.35000, 0.50000, 0.25000)	(0.46741, 0.500000, 0.250000)
C 2	(0.65000, 0.50000, 0.75000)	(0.53259, 0.500000, 0.750000)

Phonon dispersion

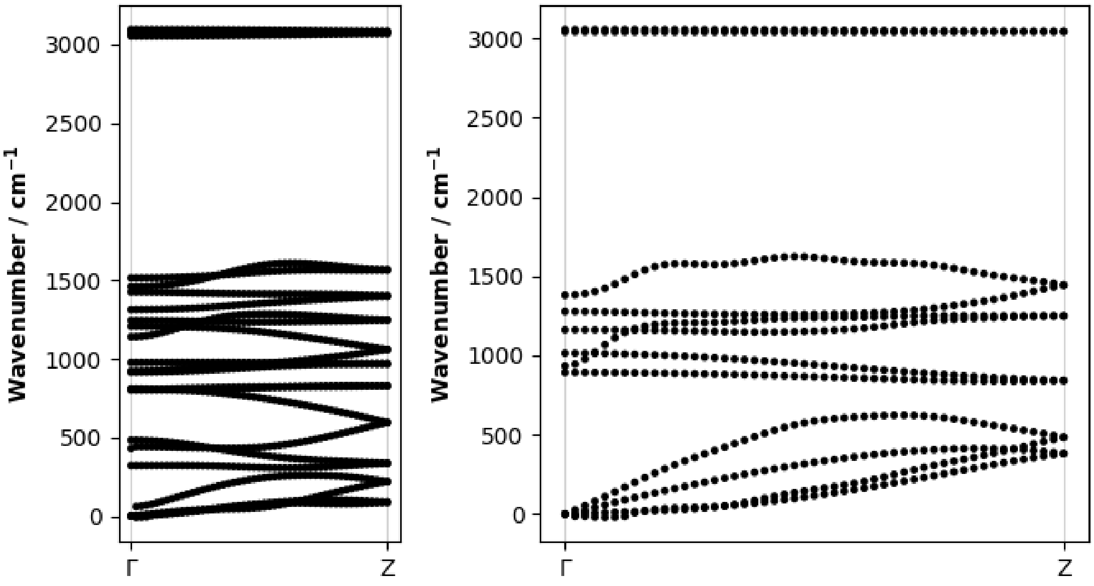

Figure S4.1: Phonon dispersion computed for (left) ***cis*-polyacetylene** and (right) ***trans*-polyacetylene**, using methods and parameters described in the main text.

Internal coordinates used for LMA

Table S4.3: Bond internal coordinates used for wavevector-resolved LMA of ***cis*-polyacetylene** as described in the main text. We note that internal coordinate pairs (1, 2) and (3, 4) are equivalent by symmetry, therefore there are two unique C–C bond force constants for this system.

Index	Atom κ_1	Cell $\mathbf{R}_1$	Atom κ_2	Cell $\mathbf{R}_2$	Bond length (Å)
1	C 1	(0,0,0)	C 2	(0,0,0)	1.4117056
2	C 3	(0,0,0)	C 4	(1,0,0)	1.4117056
3	C 2	(0,0,0)	C 3	(0,0,0)	1.3887355
4	C 4	(0,0,0)	C 1	(0,0,1)	1.3887355

Table S4.4: Bond internal coordinates used for wavevector-resolved LMA of ***trans*-polyacetylene** as described in the main text. We note that internal coordinates 1 and 2 are equivalent by symmetry, therefore there is only one unique C–C bond force constant for this system.

Index	Atom κ_1	Cell $\mathbf{R}_1$	Atom κ_2	Cell $\mathbf{R}_2$	Bond length (Å)
1	C 2	(0,0,0)	C 1	(0,0,1)	1.3957723
2	C 1	(0,0,0)	C 2	(0,0,0)	1.3957723

S4.2 Graphene

Input and optimized geometry

Table S4.5: Input and optimized unit cell parameters and fractional atomic coordinates for graphene. The input geometry was obtained from the Materials Project for C (mp-990448) from database version v2025.09.25.[2] A 8.52 Å vacuum layer in the z-direction ensures that the system is modeled as effectively two-dimensional.

	Input	Optimized
a (Å)	2.45617	2.46794
b (Å)	2.45617	2.46794
c (Å)	8.52380	8.52168
α (deg)	90	90
β (deg)	90	90
γ (deg)	120	120
Vol (Å^3)	44.5329	44.9496
Atom	$(a, b, c)_{\text{in}}$	$(a, b, c)_{\text{opt}}$
C 1	$(0.33333, 0.66667, 0.00000)$	$(0.33333, 0.66667, 0.00000)$
C 2	$(0.66667, 0.33333, 0.00000)$	$(0.66667, 0.33333, 0.00000)$

Phonon dispersion

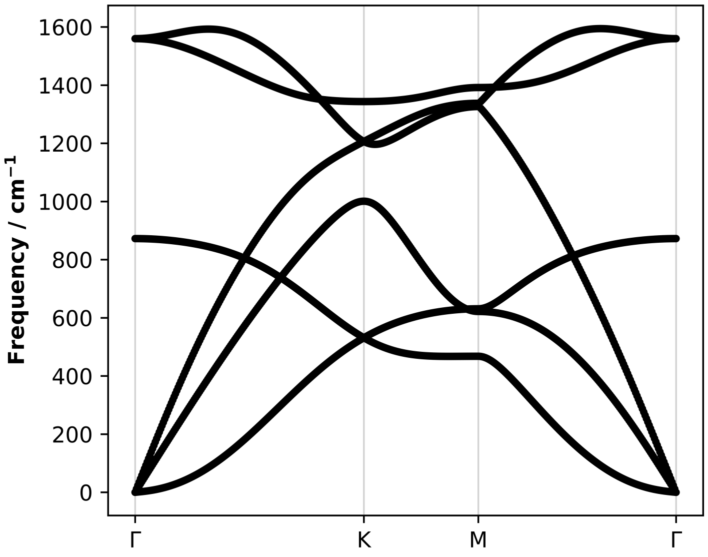

Figure S4.2: Phonon dispersion computed for graphene, using methods and parameters described in the main text.

Internal coordinates used for LMA and CNM

Table S4.6: Internal coordinates used for wavevector-resolved LMA and CNM in **graphene** as described in the main text.

**Bonds**					
Index	Atom κ_1	Cell $\mathbf{R}_1$	Atom κ_2	Cell $\mathbf{R}_2$	Bond length (Å)
1	C 1	(0,0,0)	C 2	(0,0,0)	1.4248707
2	C 2	(0,0,0)	C 1	(1,0,0)	1.4248664
**Out-of-plane buckling**					
Index	Description ($\mathbf{B}$ matrix elements)				
4	$\partial q/\partial x_{\mathrm{C1}} = (0, 0, -1)$ $\quad \partial q/\partial x_{\mathrm{C2}} = (0, 0, 1)$				
**Special translational coordinates**					
Index	Description ($\mathbf{B}$ matrix elements)				
4	$\partial q/\partial \mathbf{x}_{\mathrm{C1}} = (1, 0, 0)$ $\quad \partial q/\partial \mathbf{x}_{\mathrm{C2}} = (1, 0, 0)$				
5	$\partial q/\partial \mathbf{x}_{\mathrm{C1}} = (0, 1, 0)$ $\quad \partial q/\partial \mathbf{x}_{\mathrm{C2}} = (0, 1, 0)$				
6	$\partial q/\partial \mathbf{x}_{\mathrm{C1}} = (0, 0, 1)$ $\quad \partial q/\partial \mathbf{x}_{\mathrm{C2}} = (0, 0, 1)$				

S4.3 MgO Rock Salt

Input and optimized geometry

Table S4.7: Input and optimized unit cell parameters and fractional atomic coordinates for MgO. The input geometry was obtained from the Inorganic Crystal Structure Database (ICSD) with collection code 159375.[3]

	Input	Optimized
a (Å)	2.97197	2.97813
b (Å)	2.97197	2.97813
c (Å)	2.97197	2.97813
α (deg)	60	60
β (deg)	60	60
γ (deg)	60	60
Vol (Å^3)	18.5617	18.6774
Atom	$(a, b, c)_{\mathrm{in}}$	$(a, b, c)_{\mathrm{opt}}$
Mg	$(0.00000, 0.00000, 0.00000)$	$(0.00000, 0.00000, 0.00000)$
O	$(0.50000, 0.50000, 0.50000)$	$(0.50000, 0.50000, 0.50000)$

Phonon dispersion

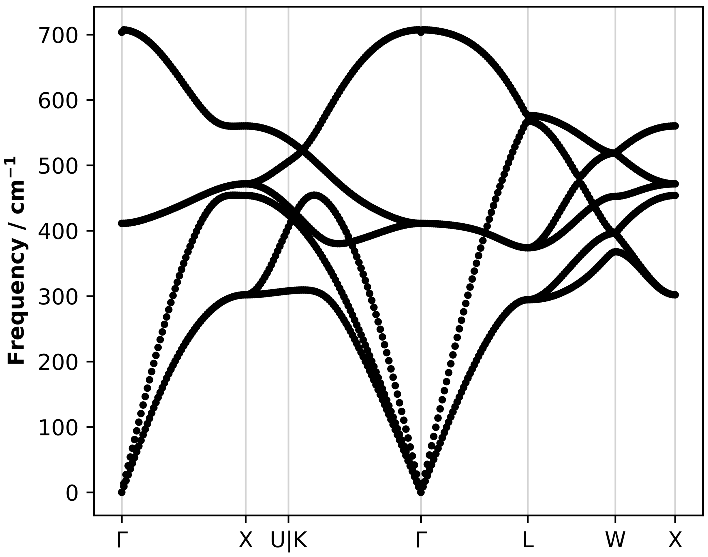

Figure S4.3: Phonon dispersion computed for MgO, using methods and parameters described in the main text.

Internal coordinates used for LMA and CNM

Table S4.8: Internal coordinates used for wavevector-resolved LMA and CNM in **MgO** as described in the main text.

**Bonds**					
Index	Atom κ_1	Cell $\mathbf{R}_1$	Atom κ_2	Cell $\mathbf{R}_2$	Bond length (Å)
1	O	(0,0,0)	Mg	(0,0,1)	2.1058552
2	O	(0,0,0)	Mg	(0,1,0)	2.1058552
3	O	(0,0,0)	Mg	(0,1,1)	2.1058552
**Special translational coordinates**					
Index	Description (**B** matrix elements)				
4	$\partial q/\partial \mathbf{x}_{\mathrm{O}} = (1,\ 0,\ 0)$ $\quad \partial q/\partial \mathbf{x}_{\mathrm{Mg}} = (1,\ 0,\ 0)$				
5	$\partial q/\partial \mathbf{x}_{\mathrm{O}} = (0,\ 1,\ 0)$ $\quad \partial q/\partial \mathbf{x}_{\mathrm{Mg}} = (0,\ 1,\ 0)$				
6	$\partial q/\partial \mathbf{x}_{\mathrm{O}} = (0,\ 0,\ 1)$ $\quad \partial q/\partial \mathbf{x}_{\mathrm{Mg}} = (0,\ 0,\ 1)$				

S4.4 $KMgF_3$ Perovskite

Input and optimized geometry

Table S4.9: Input and optimized unit cell parameters and fractional atomic coordinates for $KMgF_3$. The input geometry was obtained from the Inorganic Crystal Structure Database (ICSD) with collection code 40477.[4] We note that the optimized geometry was identical to the input geometry.

	Input	Optimized
a (Å)	4.01402	4.01402
b (Å)	4.01402	4.01402
c (Å)	4.01402	4.01402
α (deg)	90	90
β (deg)	90	90
γ (deg)	90	90
Vol (Å^3)	64.6755	64.6755
Atom	$(a, b, c)_{\mathrm{in}}$	$(a, b, c)_{\mathrm{opt}}$
F 1	$(0.00000, 0.00000, 0.50000)$	$(0.00000, 0.00000, 0.50000)$
F 2	$(0.00000, 0.50000, 0.00000)$	$(0.00000, 0.50000, 0.00000)$
F 3	$(0.50000, 0.00000, 0.00000)$	$(0.50000, 0.00000, 0.00000)$
Mg	$(0.00000, 0.00000, 0.00000)$	$(0.00000, 0.00000, 0.00000)$
K	$(0.50000, 0.50000, 0.50000)$	$(0.50000, 0.50000, 0.50000)$

Phonon dispersion

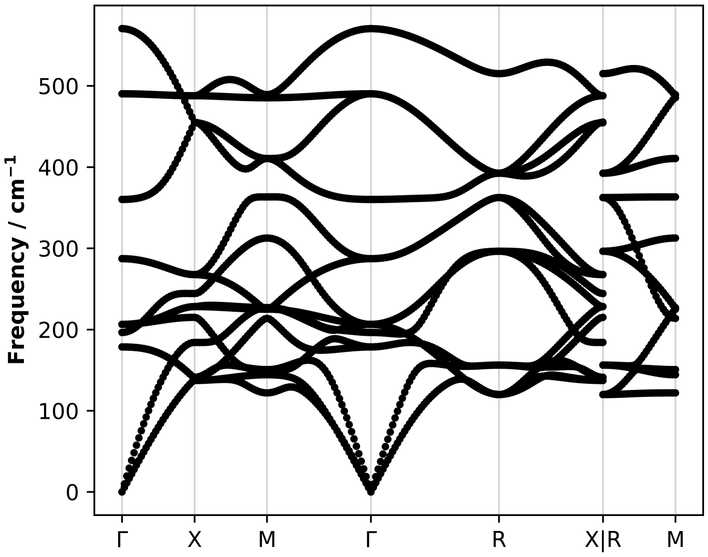

Figure S4.4: Phonon dispersion computed for $KMgF_3$, using methods and parameters described in the main text.

Internal coordinates used for LMA and CNM

For this system, we have performed LMA and CNM using an internal coordinate set generated automatically using the graph-theoretic search algorithm, including bonds, angles and dihedrals. Three additional translational coordinates were included to complete the basis for rigid-body motion. To ensure linear independence, the set was reduced using QR decomposition, retaining coordinates that contributed most significantly to the rank of the **B** matrix. The final set of internal coordinates is summarized in Table S4.10.

Additionally, we computed CNM amplitudes, $A_{n,\mu}$ (Eq. 10 in the main text), between the normal modes of $KMgF_3$ and the local modes describing the tilting of the MgF_6 octahedra. For these coordinates, each **B**-matrix element corresponds to the derivative of the internal coordinate with respect to an infinitesimal rotation of the octahedron about an axis through

its centroid, aligned with one of the Cartesian axes x, y, or z, which are given by

$$\mathbf{B}_{\alpha,R_x} = \begin{pmatrix} 0 \\ -d_{\alpha z} \\ d_{\alpha y} \end{pmatrix} \mathbf{B}_{\alpha,R_y} = \begin{pmatrix} d_{\alpha z} \\ 0 \\ -d_{\alpha x} \end{pmatrix} \mathbf{B}_{\alpha,R_z} = \begin{pmatrix} -d_{\alpha y} \\ d_{\alpha x} \\ 0 \end{pmatrix} \tag{S4.1}$$

where $d_{\alpha x}$, $d_{\alpha y}$, $d_{\alpha z}$ represent the x, y and z components of the displacement of each atom in the octahedron from the octahedron's centroid. R_x, R_y and R_z label rotations around the x, y and z axes, respectively.

Table S4.10: Internal coordinates used for wavevector-resolved LMA and CNM in $\mathbf{KMgF_3}$ as described in the main text.

Bonds

Index	Atom κ_1	Cell $\mathbf{R}_1$	Atom κ_2	Cell $\mathbf{R}_2$	Bond length (Å)
1	F 1	(0,0,0)	K	(0,0,0)	2.8383428
2	K	(0,0,0)	F 1	(0,1,0)	2.8383428
3	F 3	(0,0,0)	K	(0,0,0)	2.8383428
4	K	(0,0,0)	F 3	(0,0,1)	2.8383428
5	K	(0,0,0)	F 2	(0,0,1)	2.8383428
6	F 2	(0,0,0)	K	(0,0,0)	2.8383428
7	F 1	(0,0,0)	Mg	(0,0,0)	2.0070114
8	F 2	(0,0,0)	Mg	(0,0,0)	2.0070114
9	F 3	(0,0,0)	Mg	(0,0,0)	2.0070114

Angles

Index	Atom κ_1	Cell $\mathbf{R}_1$	Atom κ_2	Cell $\mathbf{R}_2$	Atom κ_3	Cell $\mathbf{R}_3$	Angle (deg)
10	F 3	(−1,0,0)	Mg	(0,0,0)	F 1	(0,0,0)	90
11	F 3	(−1,0,0)	Mg	(0,0,0)	F 1	(0,0,−1)	90

Dihedral

Index	Atom κ_1 Cell $\mathbf{R}_1$	Atom κ_2 Cell $\mathbf{R}_2$	Atom κ_3 Cell $\mathbf{R}_3$	Atom κ_4 Cell $\mathbf{R}_4$	Dihedral angle (deg)
12	Mg (0, 0, 0)	F 1 (0,0,0)	K (0,−1,0)	F2 (0,−1,−1)	215.2643897

Special translational coordinates

Index	Description ($\mathbf{B}$ matrix elements)	
13	$\partial q/\partial \mathbf{x}_{\mathrm{O}} = (1, 0, 0)$	$\partial q/\partial \mathbf{x}_{\mathrm{Mg}} = (1, 0, 0)$
14	$\partial q/\partial \mathbf{x}_{\mathrm{O}} = (0, 1, 0)$	$\partial q/\partial \mathbf{x}_{\mathrm{Mg}} = (0, 1, 0)$
15	$\partial q/\partial \mathbf{x}_{\mathrm{O}} = (0, 0, 1)$	$\partial q/\partial \mathbf{x}_{\mathrm{Mg}} = (0, 0, 1)$

S5 Bond order analysis under local mode distortions in *cis*- and *trans*-polyacetylene

In the main text, we show that the C–C bond stretching local mode force constants change as a function of wavevector in both *cis*- and *trans*-polyacetylene. These force constants are smaller at the Γ-point, and larger at the Z-point, indicating that the distortion patterns caused by vibrations along these local modes result in greater restoring forces when the vibrations are out-of-phase in neighboring unit cells ($\mathbf{k} = Z$). The changing local mode frequencies, which mimic the behavior of phonon branches with C–C bond stretching character, can be understood in terms of the effect of local mode distortions on the conjugation in the polymer system. Specifically, Γ-point local mode distortions preserve the conjugation pattern, while Z-point vibrations disrupt it, Figure S5.1.

To support this understanding, we performed Mayer bond order analysis on short (four repeat units) sections of the polymer chain across a range of displacements along the local mode adiabatic vectors corresponding to the C–C bond stretches. These calculations were performed using density functional theory as implemented in ORCA 6.0.0,[5] employing the PBE generalized-gradient approximation functional[6] with Grimme's D2 dispersion correction[7] and the def2-TZVP basis set. Self-consistent-field convergence criteria were set to *tight*. Our analysis shows that, for both isomers, the same change to the bond order incurs a larger energy penalty under $\mathbf{k} = Z$ displacement patterns as compared to $\mathbf{k} = \Gamma$, Figure S5.2. This is consistent with the larger Z-point local mode force constants obtained from our LMA calculations.

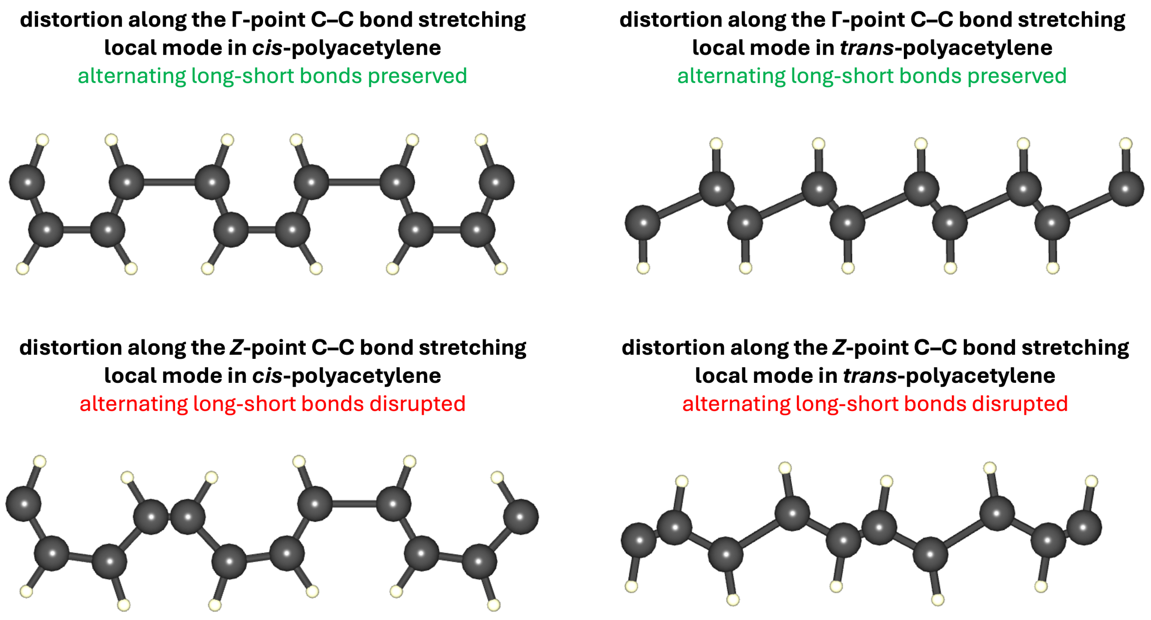

Figure S5.1: Distortion patterns resulting from C–C bond stretching local modes in *cis*- and *trans*-polyacetylene. Displacement amplitudes are exaggerated to clearly demonstrate the distortion patterns. Three repeating units are shown for *cis*-polyacetylene, and five for *trans*-polyacetylene.

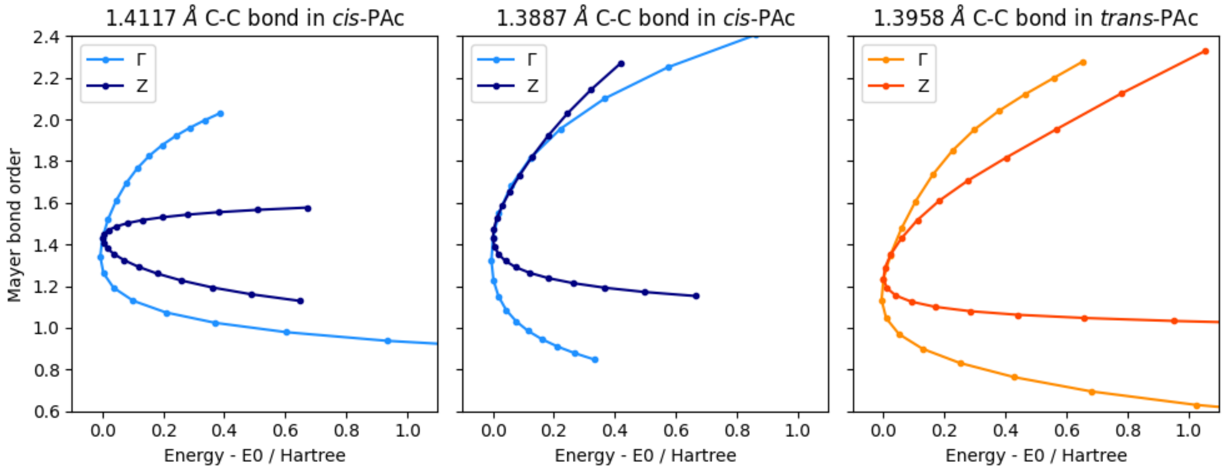

Figure S5.2: Mayer bond order vs energy change relative to the undistorted structure when small polymer sections of *cis*- and *trans*-polyacetylene are distorted along the C–C bond stretching adiabatic vectors.

References

(1) Moura, R. T. J.; Quintano, M.; Antonio, J. J.; Freindorf, M.; Kraka, E. Automatic Generation of Local Vibrational Mode Parameters: From Small to Large Molecules and QM/MM Systems. **2022**, *126*, 9313–9331.

(2) LBNL Materials Project Materials Data on C by Materials Project. 2020; `https://www.osti.gov/biblio/1316964`.

(3) Hazen, R. M. Effects of temperature and pressure on the cell dimension and X-ray temperature factors of periclase. *American Mineralogist* **1976**, *61*, 266–271.

(4) Muradyan, L. A.; Zavodnik, V. E.; Makarova, I. P.; Aleksandrov, K. S.; Simonov, V. I. Thermal vibrations of atoms in the structure of KMgF3 at 293 and 123 K. **1984**, *29*, 392–394.

(5) Neese, F. The ORCA program system. *WIRES Comput. Molec. Sci.* **2012**, *2*, 73–78.

(6) Perdew, J. P.; Burke, K.; Ernzerhof, M. Generalized Gradient Approximation Made Simple. *Phys. Rev. Lett.* **1996**, *77*, 3865–3868.

(7) Grimme, S. Semiempirical GGA-type density functional constructed with a long-range dispersion correction. *J. Comp. Chem.* **2006**, *27*, 1787–1799.
